# High Open‐Circuit Voltage Wide‐Bandgap Perovskite Solar Cell with Interface Dipole Layer

**DOI:** 10.1002/smll.202404784

**Published:** 2024-08-29

**Authors:** Jihyeon Heo, Juan Anthony Prayogo, Seok Woo Lee, Hansol Park, Senthilkumar Muthu, JeeHee Hong, Haeun Kim, Young‐Hoon Kim, Dong Ryeol Whang, Dong Wook Chang, Hui Joon Park

**Affiliations:** ^1^ Department of Organic and Nano Engineering Hanyang University Seoul 04763 Republic of Korea; ^2^ Human‐Tech Convergence Program Hanyang University Seoul 04763 Republic of Korea; ^3^ Department of Industrial Chemistry and CECS Research Institute Pukyong National University Busan 48513 Republic of Korea; ^4^ Department of Energy Engineering Hanyang University Seoul 04763 Republic of Korea; ^5^ Department of Advanced Materials Hannam University Daejeon 34054 Republic of Korea; ^6^ Department of Semiconductor Engineering Hanyang University Seoul 04763 Republic of Korea

**Keywords:** perovskite solar cell, interface dipole layer, interfacial engineering, charge transport

## Abstract

Wide‐bandgap perovskite solar cells (PSCs) with high open‐circuit voltage (*V*_oc_) represent a compelling and emerging technological advancement in high‐performing perovskite‐based tandem solar cells. Interfacial engineering is an effective strategy to enhance *V*_oc_ in PSCs by tailoring the energy level alignments between the constituent layers. Herein, n‐type quinoxaline‐phosphine oxide‐based small molecules with strong dipole moments is designed and introduce them as effective cathode interfacial layers. Their strong dipole effect leads to appropriate energy level alignment by tuning the work function of the Ag electrode to form an ohmic contact and enhance the built‐in potential within the device, thereby improving charge‐carrier transport and mitigating charge recombination. The organic interfacial layer‐modified wide‐bandgap PSCs exhibit a high *V*_oc_ of 1.31 V (deficit of <0.44 V) and a power conversion efficiency (PCE) of 20.3%, significantly improved from the device without an interface dipole layer (*V*_oc_ of 1.26 V and PCE of 16.7%). Furthermore, the hydrophobic characteristics of the small molecules contribute to improved device stability, retaining 95% of the initial PCE after 500 h in ambient air.

## Graphical abstract

Quinoxaline‐phosphine oxide‐based molecules with strong dipole moments are introduced as cathode interfacial layers of perovskite solar cells (PSCs), enhancing charge‐carrier transport and mitigating charge recombination. The modified wide‐bandgap PSCs exhibit a high *V*_oc_ of 1.31 V and an efficiency of 20.3%, with superior stability, retaining 95% of initial efficiency after 500 h in air, which demonstrate promise for tandem cells.

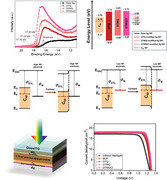

## 1 Introduction

Organic metal halide perovskite solar cell (PSC) has emerged as a promising and cost‐effective photovoltaic (PV) technology in recent years, reaching power conversion efficiency (PCE) comparable to the conventional crystalline silicon(c‐Si)‐based solar cells. Significant research progress has been made in highly efficient PSCs, facilitated by their facile fabrication methods and exceptional optoelectronic properties, including high charge‐carrier mobilities, tunable bandgaps ranging from 1.2 to 3.0 eV, high absorption coefficient, and direct bandgap.^[^ ^1^, ^2^, ^3^, ^4^, ^5^ ^]^

These beneficial characteristics of perovskites have led to substantial improvements in PCEs, surging from 3.8% to 26.1% within a decade, approaching the theoretical PCE limits estimated by Shockley and Quessier.^[^ ^6^, ^7^, ^8^, ^9^ ^]^ To overcome the theorical limit of single‐junction solar cells, research into tandem solar cells has been actively pursued, involving the integration of appropriate wide‐bandgap (*E*_g_ ≈1.8 eV) top cells and narrow‐bandgap (*E*_g_ ≈1.2 eV) bottom cells.^[^ ^10^, ^11^ ^]^ However, wide‐bandgap PSCs often encounter large open‐circuit voltage (*V*_oc_) deficits, hindering their application in highly efficient tandem solar cells.^[^ ^12^, ^13^ ^]^ In particular, the interface engineering requires thorough investigation to mitigate *V*_oc_ losses, as interfacial characteristics directly impact carrier extraction/injection and recombination dynamics.^[^ ^14^, ^15^, ^16^ ^]^

Among various configurations of PSC devices, the inverted p‐i‐n structure has been extensively studied due to its solution‐based low‐temperature processability, negligible hysteresis effect, and ease of integration with other PV technologies.^[^ ^17^, ^18^ ^]^ However, it is widely recognized that a significant energy level mismatch exists between the lowest unoccupied molecular orbital (LUMO) of electron transport layers (ETLs) and the work function of the metal electrodes.^[^ ^19^ ^]^ This energy level mismatch creates an interfacial barrier, impeding the transportation of charge carriers and consequently leading to reduced PCEs aroused by energy loss (*E*_loss_) at the interface.^[^ ^20^, ^21^, ^22^, ^23^, ^24^ ^]^ To address this limitation, modification of the ETL/metal electrode interface has been widely explored, employing thin interfacial layers such as bathocuproine (BCP), LiF, ZnO, etc.^[^ ^25^, ^26^, ^27^, ^28^, ^29^ ^]^ Furthermore, the utilization of appropriate interfacial layers could significantly enhance the device performance, particularly in reducing the contact resistance or current leakage, both of which are closely related to the *V*_oc_, a crucial PV parameter determining the PCE of solar cells.^[^ ^30^, ^31^ ^]^ Additionally, the interfacial layers can function as buffer layers, shielding the ETL and perovskite layers from the detrimental factors during the metal electrode deposition.^[^ ^32^ ^]^

Organic molecules have been widely utilized as efficient interfacial layers at various interfaces within the device architecture of PSCs for realizing high efficiency and stability.^[^ ^33^, ^34^, ^35^ ^]^ Among the wide array of organic molecules, BCP has particularly gained prominence in high‐performance PSCs, as a thin BCP layer facilitates electron extraction through the tunneling effect.^[^ ^21^, ^29^ ^]^ However, it has been reported that the BCP layer is susceptible to rapid crystallization in moist conditions, leading to instability in PSCs.^[^ ^32^ ^]^ Therefore, the search for alternative and effective interfacial layers that can facilitate the electron transportation even with superior stability has gained significant interest in recent years. Recent investigations have explored n‐type quinoxaline‐phosphine oxide‐based small molecules as interfacial layers, effectively enhancing charge transport and improving device stability through the substitution of various functional groups.^[^ ^36^, ^37^, ^38^ ^]^

In this work, three quinoxaline‐phosphine oxide‐based small molecular interfacial layers are designed for introduction between the organic ETL (C_60_) and metal electrode (Ag) interface as alternative interfacial layers to the conventional BCP layer. These molecules contain multiple functional groups, including thiophene, methoxy, and chlorine units, which provide asymmetric chemical structures and thus increase intrinsic dipole moments.^[^ ^39^ ^]^ The designed interfacial layers, with increased dipole moments, are confirmed to be highly effective in reducing the apparent work function of the cathode to form ohmic contact and enhance the built‐in potential, thereby improving charge‐carrier transport and mitigating charge recombination. Consequently, highly efficient wide‐bandgap PSCs with a high *V*_oc_ exceeding 1.31 V (*V*_oc_ deficit of <0.44 V) and a high PCE of 20.3% are fabricated using a small molecule‐based cathode interfacial layer. Furthermore, the superior stability of the inverted PSCs employing the interfacial layer on the ETL is also confirmed.

## 2 Results and Discussion

### 2.1 Design of Organic Molecules

The work function of the metal cathode can be effectively tuned by employing an interfacial layer with high intrinsic dipole moments at the interface with the ETL. When molecules with sufficiently high intrinsic dipole moments are subjected to the built‐in electric field within the p‐i‐n PSCs, strong induced dipole moments (polarizability × electric field) are generated, which point toward the perovskite surface (e.g., positive at the perovskite‐side).^[^ ^40^ ^]^ These induced dipoles decrease the work function of the metal cathode, consequently increasing the built‐in potential within the device, which is advantageous for charge transport and reduces recombination (Figure S1, Supporting Information). Furthermore, the reduced work function of the cathode metal electrodes is also beneficial for forming ohmic contact with the C_60_ ETL, whereas the ETL and metal contact without an interfacial layer is prone to forming Schottky junction.^[^ ^21^, ^41^, ^42^, ^43^ ^]^ All these aspects will be discussed in the next section.

**Figure 1:**
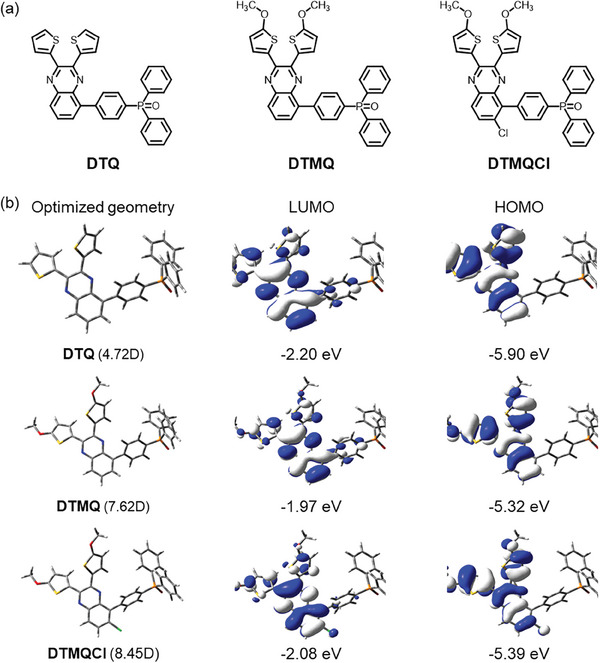
a) Molecular structure of DTQ, DTMQ, and DTMQCl. b) Optimized geometry and dipole moment with electron density plot at HOMO and LUMO energy levels at B3LYP/6‐31G**.

Incorporating the phosphine oxide group (P = O) into the electron‐accepting quinoxaline unit effectively enhances its dipole characteristics. Additionally, it can increase the solubility of the molecule in polar solvents such as isopropyl alcohol, commonly used for the fabrication of inverted PSCs, consequently improving its processability.^[^ ^36^, ^44^ ^]^ Meanwhile, thiophene, a commonly used heterocyclic compound, was further introduced at the 2, 3 positions of the quinoxaline unit to further tune and optimize the molecular dipole moment. The introduction of thiophene rings also allows the designed molecule to exhibit outstanding charge transport properties and significantly adjust its energy levels.^[^ ^45^, ^46^ ^]^ This results in the synthesis of the first molecule, (4‐(2,3‐di(thiophen‐2‐yl)5uinoxaline‐5‐yl)phenyl)diphenylphosphine oxide (DTQ). To further enhance the dipole moment, electron‐donating methoxy unit (‐OCH_3_) substituents were incorporated into the thiophene 5 positions, significantly enhancing the dipole moment by strengthening push‐pull molecular interaction.^[^ ^38^ ^]^ This leads to (4‐(2,3‐bis(5‐methoxythiophen‐2‐yl)5uinoxaline‐5‐yl)phenyl)diphenylphosphineoxide (DTMQ). The molecular dipole moment can be further enhanced by introducing the C‒Cl bond,^[^ ^47^ ^]^ and this provides (4‐(6‐chloro‐2,3‐bis(5‐methoxythiophen‐2‐yl)5uinoxaline‐5‐yl)phenyl) diphenylphosphine oxide (DTMQCl). Chlorination of n‐type materials has been an effective strategy to improve the charge carrier mobility and long‐term stability in optoelectronic devices.^[^ ^48^, ^49^, ^50^ ^]^ This push‐pull effect creates a more efficient charge separation and transport mechanism. This is a novel approach in the context of quinoxaline‐phosphine oxide‐based small molecules. Furthermore, the design strategy involving thiophene side chains, methoxy and chlorine functional moieties, and phosphine oxide groups can be easily adapted to other types of organic interfacial materials to tune its optoelectronic properties. This demonstrates its versatility and potential for widespread application in different device systems. The chemical structures of newly designed small molecules are depicted in **Figure** **1**a, and the detailed synthetic procedures are provided in Figure S2 (Supporting Information). The chemical structures of the prepared small molecules were further verified through the mass spectroscopic techniques and nuclear magnetic resonance spectroscopy (NMR) (Figures S3–S14, Supporting Information).

The frontier molecular orbitals, optimized geometries, and molecular dipole moment values of DTQ, DTMQ, and DTMQCl were investigated through density functional theory (DFT) calculations using the Gaussian model at the B3LYP/6‐31G** level (Figure 1b).^[^ ^51^ ^]^ The calculated molecular dipole moments of the DTQ, DTMQ, and DTMQCl were estimated to be 4.72, 7.62, and 8.45 D, respectively, as designed. Figure 1b shows that the electron delocalization mainly occurs on the thiophene and quinoxaline units at both the LUMO and highest occupied molecular orbital (HOMO) levels. The simulated HOMO/LUMO energy levels for DTQ, DTMQ, and DTMQCl were −5.90/−2.20, −5.32/−1.97, and −5.39/−2.08, respectively. It has been identified that the addition of an electron‐donating methoxy unit to DTQ increases its HOMO/LUMO energy levels, as well as the dipole moment. Furthermore, the electron‐withdrawing Cl in the DTMQCl further increased the dipole moment but slightly reduced the HOMO/LUMO energy levels compared to DTMQ.

**Figure 2:**
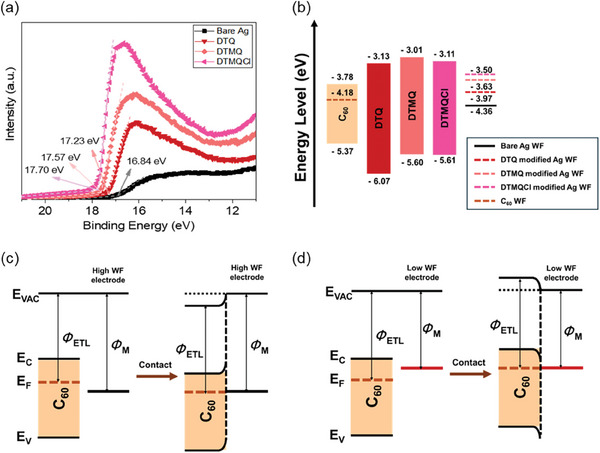
a) Ultraviolet photoelectron spectroscopy (UPS) binding energy profiles representing the secondary electron cut‐off energy values associated with the work functions of bare Ag and Ag layers on DTQ, DTMQ, and DTMQCl. b) Energy level diagrams of C_60_, DTQ, DTMQ, and DTMQCl. Schematic figures of band bending of C_60_ contacted with c) high work function Ag forming Schottky barrier and d) low work function Ag forming ohmic contact.

The UV–vis absorption spectra of the designed small molecules are illustrated in Figure S15a (Supporting Information). DTQ, DTMQ, and DTMQCl exhibit similar absorption characteristics in the wavelength range of 300 to 500 nm, with two distinct absorption peaks. The peak located at 308 – 330 nm corresponds to *π*–π* transition, while the second absorption peak located at 395–445 nm arises from the intramolecular charge transfer (ICT).^[^ ^52^ ^]^ However, compared to DTQ, the absorption peaks for DTMQ and DTMQCl are red‐shifted, attributed to the influence of their respective substituents. In particular, the electron‐donating methoxy unit significantly enhances the ICT by extending π‐conjugation, thereby raising the HOMO energy level. This, in turn, decreases the energy required for electronic transitions, leading to a red‐shift in the absorption spectrum. Moreover, the bandgap of DTMQCl is further reduced due to the addition of electron‐withdrawing Cl atom. The inductive effect of the Cl atom lowers the energy levels of both LUMO and HOMO by increasing electron affinity; however, this effect is often more pronounced on the LUMO than on the HOMO, especially when the electron‐withdrawing Cl substituent is incorporated into the acceptor unit of D‐A type polymers.^[^ ^53^, ^54^ ^]^ Consequently, the addition Cl atom leads to a reduced bandgap. The estimated bandgap values are 2.75, 2.48, and 2.44 eV for the DTQ, DTMQ, and DTMQCl, respectively. The LUMO energy levels of DTQ, DTMQ, and DTMQCl were measured to be −3.63, −3.60, and −3.61 eV, respectively, using cyclic voltammetry (Figure S15b, Supporting Information). The HOMO energy levels of these molecules were calculated from the corresponding LUMO energy levels and optical bandgap, resulting in values of −6.38, −6.08, and −6.05 eV for DTQ, DTMQ, and DTMQCl, respectively. The estimated energy levels of the designed molecules are depicted in **Figure** **2**b.

### 2.2 Charge Transfer Dynamics

To verify the influence of interfacial layers on the work function of the metal electrode (Ag in this work), we conducted ultraviolet photoelectron spectroscopy (UPS) analysis and the Kelvin probe force microscope (KPFM) analysis, as shown in Figure 2a, Figures S16 and S17 (Supporting Information). The work functions of Ag electrodes with and without the interfacial layers were obtained from the secondary electron cut‐off region in UPS spectra.^[^ ^43^, ^55^ ^]^ The work function of the pristine C_60_ layer was estimated as 4.18 eV (Figure S18, Supporting Information), while that of Ag without interfacial layer was 4.36 eV. Thus, the work function of Ag, higher than that of C_60_ (Figure 2b,c), could cause the formation of a Schottky barrier between the ETL and the metal electrode, leading to significant charge accumulation and non‐radiative recombination, resulting in *E*_loss_ at the interface.^[^ ^56^, ^57^ ^]^ In contrast, the work functions of Ag contacts with the interfacial layers decreased to 3.97, 3.63, and 3.5 eV for DTQ, DTMQ, and DTMQCl, respectively, resulting in the formation of ohmic contacts (Figure 2d). Furthermore, as shown in Figures S16 and S17 (Supporting Information), the average surface potential of Ag without interfacial layer and Ag with organic interfacial layers (DTQ, DTMQ, and DTMQCl) are −173.74, 105.46, 470.57, and 579.67 mV, respectively, and the corresponding work function are estimated to be 4.84, 4.57, 4.20, and 4.09 eV. The trends of KPFM measurement results are consistent with UPS results, further verifying the efficient regulation of work function of Ag electrode with the organic interfacial layers. Consequently, the effect of interfacial layers on reducing the Ag work function facilitates efficient charge transport, which is a key factor closely related to device performance, and this effect is maximized with the DTMQCl, which has the highest dipole moment.

**Figure 3:**
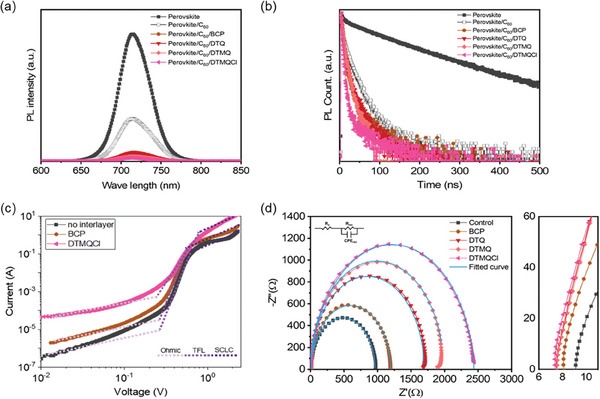
a) Steady‐state photoluminescence (PL) spectra and b) time‐resolved photoluminescence (TRPL) decay curves of perovskite films having different configurations. c) Dark *J–V* curves of the electron‐only devices (ITO/SnO_2_/perovskite/C_60_/interfacial layer/Ag) with and without interfacial layers. d) Nyquist plots of the devices with and without interfacial layers.

To investigate the charge transfer behavior of at the cathode‐side interface with and without the interfacial layers, the steady‐state photoluminescence (PL) analysis was conducted. As shown in **Figure** **3**a, the PL intensity of the perovskite layer was quenched in the presence of the C_60_ layer, indicating charge transfer from the perovskite to the C_60_ ETL. Furthermore, the perovskite layers featuring additional interfacial layers at the interface with the C_60_ exhibited a further reduction in PL intensity, with decreasing trend correlated to the strength of dipole effect–the lowest PL intensity with the DTMQCl. We also performed time‐resolved photoluminescence (TRPL) measurements for further analysis of the charge extraction characteristics with the interfacial layers (Figure 3b). The PL decay lifetimes of the perovskite films with and without the interfacial layers were obtained by fitting the TRPL spectra following a biexponential Equation (1) (Figure 3b; and Table S1, Supporting Information):^[^ ^58^, ^59^ ^]^

(1) $$\tau_{avg}=A_{1}\exp\left(-\frac{t}{\tau_{1}}\right)+A_{2}\exp\left(-\frac{t}{\tau_{2}}\right)$$

where *τ*_1_ and *τ*_2_ are the lifetimes for the slow and fast decay components, respectively, and *A*_1_, and *A*_2_ are their relative amplitude. The average PL lifetimes (*τ*_avg_) of the perovskite films were decreased with interfacial layers compared with the pristine perovskite (84.12 ns) and perovskite/C_60_ samples (6.43 ns), and the detailed parameters are presented in Table S1 (Supporting Information). Especially, the DTMQCl‐modified sample exhibited the fastest *τ*_avg_ of 2.34 ns compared to that with DTQ (4.71 ns) and DTMQ (4.68 ns). The decreased PL lifetimes of the perovskite with interface modification support more efficient charge extraction, which could be attributed to the influence of interfacial layers.

Furthermore, the impact of the interfacial layer on charge transport behaviors was further assessed by analyzing the space‐charge‐limited current (SCLC) of electron‐only devices (ITO/SnO_2_/perovskite/C_60_/interfacial layer/Ag) (Figure 3c; Figure S19, Supporting Information) using the Mott‐Gurney's law (2):^[^ ^60^, ^61^, ^62^ ^]^

(2) $$J=\frac{9\varepsilon \varepsilon_{0}\mu V^{2}}{8L^{3}}\;$$

where, *ε*, *ε*_o_, and *L* represent the relative permittivity, the vacuum permittivity, and the thickness of the perovskite film, respectively. By fitting the curves in the SCLC region, the electron mobilities (*µ*) of devices were calculated, resulting in values of 1.84 × 10^−2^ and 4.77 × 10^−2^ cm^2 ^V^−1 ^s^−1^ for BCP and DTMQCl‐based devices, respectively, while 6.61 × 10^−3^ cm^2 ^V^−1 ^s^−1^ for the device without interfacial layer). All interfacial layers enhanced carrier transport in the device, with the most pronounced effect observed with the DTMQCl, featured by the highest dipole moment, which is consistent with the results obtained from PL and TRPL analyses.

The charge transfer and recombination kinetics were further confirmed by electrochemical impedance spectroscopy (EIS) (Figure 3d). The impedance Z can be interpreted by following equation:

(3) $$Z=Z_{0}\;\left(\cos\left(\varphi \right)-i\sin\left(\varphi \right)\right)=Z^{\prime }\;-iZ^{\prime \prime }$$

where Z is a complex function given by $Z=\sqrt{\left({Z^{\prime }}^{2}+{Z^{\prime \prime }}^{2}\right)}$ and *φ* is the phase angle. In response to this relation, the real‐axis intersection (*φ* = 0°) of the semicircle indicates resistance behavior, while the imaginary axis (*φ* = – 90°) corresponds to the capacitance behavior.^[^ ^63^ ^]^ It is generally accepted that the high frequency region generally corresponds to the series resistance (*R_s_*), while those in the low frequency region result from the recombination resistance (*R_rec_*). Since the capacitance behavior is negligible in the high frequency region, only the resistance value (*R_s_*) can be observed. As the frequency decrease, the capacitance value increases, and as the frequency approaches to zero, the reactance also converges, leaving only the resistance value (*R_rec_*). Each resistance value was evaluated using equivalent circuit model in the inset of Figure 3d.^[^ ^20^, ^63^, ^64^, ^65^, ^66^, ^67^ ^]^ As shown in Figure 3d and Table S2 (Supporting Information), the *R*_s_ values decrease and the *R*_rec_ values increase for the devices with interfacial layer modification. In particular, the device with the DTMQCl interlayer exhibits the highest *R*_rec_ value of 2463.4 Ω and the lowest *R*_s_ value of 7.4 Ω. The device without interlayer shows *R*_rec_ and *R*_s_ values as 975.4 and 14.1 Ω, respectively. The obtained results imply the enhanced charge transfer and reduced recombination at the cathode interface with the interlayer.

### 2.3 Photovoltaic Performance of PSCs

**Figure 4:**
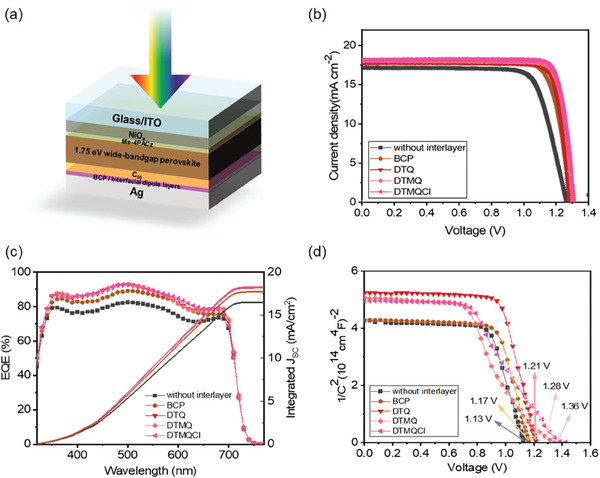
a) Graphical representation of perovskite solar cell (PSC) architecture. b) *J*–*V* characteristics of the PSC devices (forward scan), with and without interfacial layers (simulated AM 1.5G at 100 mW cm^−2^). c) EQE spectra with integrated photocurrent of the PSC devices, with and without interfacial layers. d) Mott‐Schottky plots of the PSC devices, with and without interfacial layers.

The enhanced charge transport and extraction characteristics at the cathode interface with the designed interfacial layers result in improved performance of the p‐i‐n wide bandgap PSCs (ITO/NiO_x_/Me‐4PACz/perovskite/C_60_/interfacial layer/Ag) (**Figure** **4**a). Figure 4b shows the current density‐voltage (*J*–*V*) characteristics of the devices with and without the interfacial layers. The performance of the PSC without the interfacial layer, which is just 16.7% PCE with 1.26 V *V*_oc_, increases to 18.8% PCE with 1.28 V *V*_oc_ after introducing the conventional BCP interfacial layer. These values are further increased with the designed interfacial layers and maximized with the DTMQCl, resulting in outstanding performance (20.3% PCE with *V*_oc_ of 1.31 V). The PV parameters of the best‐performing devices are summarized in **Table** **1**. Additionally, Figure S20 and Table S3 (Supporting Information) show that the hysteresis of the *J*–*V* curve about the scan direction in the device without the interfacial layer disappeared in the device with the DTMQCl. The hysteresis index (HI) of the DTMQCl‐treated device, defined by [{PCE(reverse)‐PCE(forward)}/PCE(reverse)], is ≈0.01, while the device without an interfacial layer exhibits larger hysteresis (HI≈0.05). It is worth noting that the enhanced performance in the device with the DTMQCl is mainly attributed to the *V*_oc_ enhancement with an impressive *V*_oc_ deficit of less than 0.44 V, which is one of the highest *V*_oc_ values of the PSCs having similar bandgap to our device in the literature (Figure S21 and Table S4, Supporting Information).

**Table 1:** Photovoltaic performances of PSCs with and without interfacial layers (DTQ, DTMQ, and DTMQCl), obtained by *J*–*V* characteristics (scanned in forward direction).

	*V*_oc_ [V]	*J*_sc_ [mA cm^−2^]	Fill Factor	PCE [%]
without interfacial layer	1.26	17.2	76.8	16.7
BCP	1.28	17.8	82.2	18.8
DTQ	1.29	18.0	84.4	19.6
DTMQ	1.30	18.1	84.5	19.9
DTMQCl	1.31	18.1	85.6	20.3

To further confirm the improved PV characteristics with the designed interfacial layers, the external quantum efficiency (EQE) of the devices was analyzed (Figure 4c). The enhanced EQE with a peak value of 91% was observed for the devices with the DTMQCl, compared to 80% for the device without an interfacial layer, and the integrated *J*_sc_ value of the DTMQCl‐device is ≈18 mA cm^−2^–≈17 mA cm^−2^ for the device without an interfacial layer–which are well‐matched to the values obtained by the *J*–*V* curve. Moreover, it is noteworthy that the fill factors are also greatly improved by applying organic interfacial layer comparing to the device without interfacial layers. This increase in fill factors with the organic interfacial layers is consistent with the EIS results, as fill factor is highly related with the resistance characteristics.^[^ ^68^, ^69^ ^]^

The *V*_oc_ enhancement with interfacial layers can be further assessed by analyzing the built‐in potential (*V*_bi_) variation of the devices. The Mott‐Schottky plot (*C* ^2^‐*V*) were used to estimate the value of *V*_bi_ (Figure 4d) with the following equation:^[^ ^70^, ^71^, ^72^, ^73^ ^]^

(4) $$C^{-2}=2\left(V_{bi}-V\right){\left(A^{2}e\varepsilon \varepsilon_{0}N_{A}\right)}^{-1}$$

where *V* is the applied bias, *A* is the active device area, and *N_A_* is the doping concentration. As depicted in Figure 4d, a much higher *V*_bi_ of 1.36 V is obtained from the device with the DTMQCl, compared to the devices without an interfacial layer (1.13 V).

### 2.4 Device Stability of PSCs with Organic Interfacial Layer

**Figure 5:**
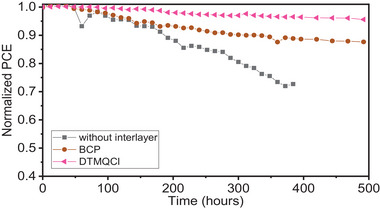
Stability of PSC devices with and without DTMQCl interfacial layer, along with that with BCP layer, and without IDLs (DTQ, DTMQ, and DTMQCl) under ambient condition with a relative humidity of 30–50%.

In addition to enhancing the performance, the interfacial layers were found to improve the stability of device due to their hydrophobic characteristics. Figure S22 (Supporting Information) shows the contact angles of pristine C_60_, and C_60_ treated with the organic films. The C_60_ thin films modified with organic layers exhibits increased hydrophobicity with estimated contact angles of 52.7°, 54.2°, and 51.3°, considerably higher than the pristine C_60_ film (31.8°). The increased hydrophobic properties of the organic‐modified film are expected to enhance the stability of the device under ambient condition. **Figure** **5** and Figure S23 (Supporting Information) represents the long‐term stability of the wide‐bandgap PSCs, performed for 500 h under ambient condition with a relative humidity of 30–50%. The devices with the interfacial layers (DTQM, DTMQ, and DTMQCl) maintained over 90% PCE of their initial PCE, while the device without interfacial layer operated for less than 400 h (Figure S23, Supporting Information). For reference, the performance of the device with BCP dropped to under 90% of its initial PCE after 500 h (Figure 5). An additional Al_2_O_3_ layer was added to the devices by atomic layer deposition (ALD) for stability test. An operational stability of the device with DTMQCl was also confirmed by maximum power point tracking (MPPT) (Figure S24, Supporting Information).

## 3 Conclusion

In summary, we have investigated the interface modification between the C_60_ ETL and Ag cathode in wide band‐gap PSCs using the designed organic interfacial layers DTQ, DTMQ, and DTMQCl to enhance device performance and improve charge carrier transport. Employing the organic molecules with strong dipole moments as interfacial layers reduces the work function of the cathode, lowering the energy level barrier to form ohmic contact and enhancing the built‐in potential. Consequently, these modifications improve charge‐carrier transport and mitigate charge recombination. Notably, the PSC device with the DTMQCl achieved a high PCE of 20.3% with a significantly reduced *V*_oc_ deficit of 0.44 V, compared to the device without an interfacial layer, which exhibited a PCE of 16.7% with a *V*_oc_ deficit of 0.49 V. Furthermore, the interface modification with the interfacial layer contributed to improved device stability, retaining 95% of its initial PCE even after 500 h, owing to the hydrophobic characteristics of the organic interfacial layers. These advancements in increased *V*_oc_, PCE and stability of wide band‐gap PSCs through interface engineering suggest potential pathways for highly efficient and stable tandem cell approaches.

## 4 Experimental Section

### Materials and Synthetic Method for Organic Small Molecules

The 2,2′‐thenil (5, Figure S2, Supporting Information) and all other reagents and solvents were purchased from Sigma‐Aldrich Co. and were used without further purification. 4‐bromo‐2,1,3benzothiadiazole (3, Figure S2, Supporting Information),^[^ ^35^ ^]^ 1,2‐bis(5‐methoxythiophen‐2‐yl)ethane‐1,2‐dione (6, Figure S2, Supporting Information),^[^ ^74^ ^]^ and diphenyl(4‐(4,4,5,5‐tetramethyl‐1,3,2‐dioxaborolan‐2‐yl)phenyl)phosphine oxide (10, Figure S2, Supporting Information)^[^ ^36^ ^]^ were prepared as described in the previous literature.

### 4‐bromo‐5‐chlorobenzo[c][1,2,5]thiadiazole (**4**)

5‐chloro[2,1,3]benzothiadiazole (2, 5 g, 29.3 mmol), synthesized according to literature procedure,^[^ ^75^ ^]^ was dissolved in 48% HBr (80 mL). Bromine (1.36 mL, 26.64 mmol) was then added dropwise to the solution and the reaction mixture was refluxed for 2 h. After quenching the reaction with NaHSO_3_, the mixture was poured into water and extracted with dichloromethane (MC). The organic layers were collected, dried over MgSO_4_, and filtered. After removal of the solvent, the crude product was purified by column chromatography on a silica gel using MC/hexane (1/4, v/v) as the eluent. Yield = 65% (white solid). 1H NMR (400 MHz, CDCl_3_) δ (ppm) = 7.91 (d, J = 9.6 Hz, 1H), 7.67 (d, J = 9.1 Hz, 1H). 13C NMR (100 MHz, CDCl_3_): δ (ppm) = 154.10, 152.71, 136.78, 131.55, 120.62, 114.28 (Figure S2, Supporting Information).

### 5‐bromo‐2,3‐di(thiophen‐2‐yl)quinoxaline (**7**)

In a round bottom flask, solution of 4‐bromo‐2,1,3benzothiadiazole (3, 1 g, 4.65 mmol) and zinc powder (69.7 mmol) were stirred in acetic acid (50 mL) for 30 min at 85 °C. After the reaction was completed, the zinc powder was filtered off. 2,2′‐thenil (5, 3.87 mmol) was then added into the filtrate and the mixture was stirred at 110 °C for overnight. After cooling down to room temperature, the reaction was poured into water and extracted with dichloromethane three times. The organic layers were collected, dried over MgSO_4_, and filtered. After removal of the solvent, the crude product was purified by column chromatography using MC/hexane (1/1, v/v) as the eluent. Yield = 76% (Light yellow solid). 1H NMR (400 MHz, CDCl_3_) δ (ppm) = 8.00–8.03 (m, 2H), 7.51‐7.57 (m, 3H), 7.37–7.39 (m, 1H), 7.33 (dd, J = 3.9, 1.1 Hz, 1H), 7.08 (dd, J = 5.0, 3.7 Hz, 1H), 7.02 (dd, J = 5.0, 3.7 Hz, 1H). 13C NMR (100 MHz, CDCl_3_): δ (ppm) = 147.09, 146.94, 141.62, 141.15, 140.61, 138.38, 133.46, 130.09, 130.07, 129.87, 129.68, 129.24, 128.55, 127.69, 127.63, 123.64 (Figure S2, Supporting Information).

### 5‐bromo‐2,3‐bis(5‐methoxythiophen‐2‐yl)quinoxaline (**8**)

A similar procedure to prepared 7 was applied to synthesized 8. 1,2‐bis(5‐methoxythiophen‐2‐yl)ethane‐1,2‐dione (6) was used as the reagent instead of 5. The crude product was purified by column chromatography using MC/hexane (1/1, v/v) as the eluent. Yield = 67% (Orange solid). 1H NMR (400 MHz, CDCl3) δ (ppm) = 7.88 (ddd, J = 8.0, 5.3, 1.1 Hz, 2H), 7.43 (t, J = 8.0 Hz, 1H), 7.34 (d, J = 4.1 Hz, 2H), 6.16 (dd, J = 13.7, 4.1 Hz, 2H), 3.98 (d, J = 1.4 Hz, 6H). 13C NMR (100 MHz, CDCl_3_): δ (ppm) = 170.96, 170.16, 146.58, 146.34, 140.57, 137.76, 132.62, 129.18, 129.03, 128.61, 128.05, 127.98, 126.92, 123.02, 105.02, 104.81, 60.35, 60.28 (Figure S2, Supporting Information).

### 5‐bromo‐6‐chloro‐2,3‐bis(5‐methoxythiophen‐2‐yl)quinoxaline (**9**)

A similar procedure to prepared 7 was applied to synthesized 9. 1,2‐bis(5‐methoxythiophen‐2‐yl)ethane‐1,2‐dione (6) was used as the reagent instead of 5 and 4‐bromo‐5‐chlorobenzo[c][1,2,5]thiadiazole (4) was used instead of 3. The crude product was purified by column chromatography using MC/hexane (1/1, v/v) as the eluent. Yield = 58% (Orange solid). 1H NMR (400 MHz, CDCl_3_) δ (ppm) = 7.80 (d, J = 8.7 Hz, 1H), 7.61 (d, J = 8.7 Hz, 1H), 7.38 (dd, J = 6.2, 4.3 Hz, 2H), 6.17 (dd, J = 13.0, 4.3 Hz, 2H), 3.99 (d, J = 2.7 Hz, 6H). 13C NMR (101 MHz, CDCl_3_): δ (ppm) = 171.40, 170.37, 146.99, 146.04, 138.66, 138.27, 135.75, 130.11, 130.08, 129.45, 128.77, 127.99, 127.85, 122.97, 105.17, 104.85, 60.37, 60.31 (Figure S2, Supporting Information).

### (4‐(2,3‐di(thiophen‐2‐yl)quinoxalin‐5‐yl)phenyl)diphenylphosphine oxide (DTQ)

In a Schlenk flask, 7 (1 mmol), diphenyl(4‐(4,4,5,5‐tetramethyl‐1,3,2‐dioxaborolan‐2‐yl)phenyl)phosphine oxide (10, 1 mmol), and Pd(PPh3)4 (5% mol) were dissolved in dry toluene (12 mL). Then 2 m K_2_CO_3_ aqueous solution was added, and nitrogen was bubbled for 15 min. The reaction mixture was heated and stirred at 90 °C for 48 h under N_2_ atmosphere. After cooling down to room temperature, the reaction mixture was poured into water and extracted with dichloromethane three times. The organic layers were collected, dried over MgSO_4_, and filtered. After removal of the solvent, the crude product was purified by column chromatography using EA/hexane (1/1, v/v) as the eluent. Yield = 53% (Yellow solid). ^1^H NMR (400 MHz, CDCl_3_) δ (ppm) = 8.07 (dd, J = 8.0, 1.6 Hz, 1H), 7.89‐7.92 (m, 2H), 7.73‐7.85 (m, 8H), 7.46‐7.57 (m, 7H), 7.40 (td, J = 4.3, 0.9 Hz, 2H), 7.18 (dd, J = 3.7, 0.9 Hz, 1H), 7.07 (dd, J = 5.0, 3.7 Hz, 1H), 6.93 (dd, J = 5.0, 3.7 Hz, 1H). ^13^C NMR (101 MHz, CDCl_3_): δ (ppm) = 146.16, 146.00, 142.70, 141.63, 140.99, 140.55, 138.75, 138.26, 133.34, 132.36, 132.30, 132.26, 132.05, 132.04, 131.81, 131.72, 131.20, 131.08, 130.60, 129.92, 129.79, 129.42, 129.13, 128.91, 128.68, 128.56, 127.75, 127.67. ^31^P NMR (162 MHz, CDCl_3_) δ (ppm) = 29.79. LC‐TOF MS: m/z calcd, 570.0989; found, 571.1062 [M^+^].

### (4‐(2,3‐bis(5‐methoxythiophen‐2‐yl)quinoxalin‐5‐yl)phenyl)diphenylphosphine oxide (DTMQ)

A similar procedure to prepared DTQ was applied to synthesized DTMQ. The prepared material 8 was used as the reagent instead of 7. The crude product was purified by column chromatography using EA/hexane (3/1, v/v) as the eluent. Yield = 49% (Orange solid). ^1^H NMR (400 MHz, CDCl_3_) δ (ppm) = 7.96 (dd, J = 8.2, 1.8 Hz, 1H), 7.84–7.88 (m, 2H), 7.74‐7.82 (m, 6H), 7.66‐7.72 (m, 2H), 7.55‐7.59 (m, 2H), 7.47‐7.51 (m, 4H), 7.34 (d, J = 4.1 Hz, 1H), 7.25 (d, J = 4.1 Hz, 1H), 6.19 (d, J = 4.1 Hz, 1H), 6.08 (d, J = 4.6 Hz, 1H), 3.99 (s, 3H), 3.89 (s, 3H). ^13^C NMR (101 MHz, CDCl_3_): δ (ppm) = 170.75, 170.08, 145.76, 145.31, 141.85, 139.71, 138.21, 137.67, 133.40, 132.35, 132.26, 132.02, 131.75, 131.64, 131.12, 131.00, 129.89, 128.99, 128.95, 128.66, 128.55, 128.43, 126.96, 104.90, 104.82, 60.48, 60.18. ^31^P NMR (162 MHz, CDCl_3_) δ (ppm) = 29.95. LC‐TOF MS: m/z calcd, 630.1201; found, 631.1275 [M^+^].

### (4‐(6‐chloro‐2,3‐bis(5‐methoxythiophen‐2‐yl)quinoxalin‐5‐yl)phenyl)diphenyl phosphine oxide (DTMQCl)

A similar procedure to prepared DTQ was applied to synthesized DTMQ. The prepared material 9 was used as the reagent instead of 7. The crude product was purified by column chromatography using EA/hexane (3/1, v/v) as the eluent. Yield = 48% (Orange solid). ^1^H NMR (400 MHz, CDCl_3_) δ (ppm) = 7.89 (d, J = 9.1 Hz, 1H), 7.74‐7.84 (m, 6H), 7.67 (d, J = 9.1 Hz, 1H), 7.56‐7.60 (m, 4H), 7.49‐7.54 (m, 4H), 7.34 (d, J = 4.1 Hz, 1H), 7.25 (d, J = 4.1 Hz, 1H), 6.19 (d, J = 4.1 Hz, 1H), 6.05 (d, J = 4.6 Hz, 1H), 3.99 (s, 3H), 3.85 (s, 3H). ^13^C NMR (101 MHz, CDCl_3_) δ (ppm) = 171.17, 170.18, 146.12, 145.12, 139.35, 139.32, 139.18, 138.18, 136.59, 133.47, 133.34, 132.39, 132.29, 132.03, 131.59, 131.47, 131.43, 131.34, 130.67, 128.94, 128.81, 128.70, 128.58, 128.54, 126.73, 104.87, 104.62, 60.48, 60.00. ^31^P NMR (162 MHz, CDCl_3_) δ (ppm) = 30.38. LC‐TOF MS: m/z calcd, 664.0811; found, 664.0818 [M].

### Materials

Cesium iodide (CsI, 99.9%), lead (II) bromide (PbBr_2_, 98%), Methylammonium bromide (MABr), bathocuproine (BCP, trace metal basis, 99.99%), Butane‐1,4‐diammonium iodide (BDAI_2_), chlorobenzene (CB, anhydrous), *N,N’*‐dimethylformamide (DMF, anhydrous), dimethyl sulfoxide (DMSO, anhydrous), and ethanol (anhydrous, ≥99.5%) were purchased from Sigma Aldrich. Lead iodide (PbI_2_, 10‐mesh beads, ultra‐dry, 99.999%) was purchased from Alfa Aesar. [4‐(3,6‐Dimethyl‐9H‐carbazol‐9‐yl)butyl] phosphonic Acid (Me‐4PACz) was purchased from TCI. Methylammonium iodide (MAI, 99.99%), formanmidinium iodide (FAI, 99.99%) and propane‐1,3‐diammonium iodide (PDAI_2_) were purchased from the GreatCell Solar Company. Fullerene (C_60_) was purchased from Nano‐C Corp. Silver pellets (Ag, 99.99%) was purchased from RND Korea. The synthesis of NiO_x_ nanoparticles (NPs) were followed by our previous work.^[^ ^14^ ^]^

### Preparation of Perovskite Precursor Solution

Perovskite solution (1.2 m) was prepared followed by the composition of FA_0.80_MA_0.04_Cs_0.16_Pb(I_0.68_Br_0.32_)_3_ by mixing FAI, MAI, CsI, PbI_2_ and PbBr_2_ in a stoichiometric ratio with a mixed solvent of DMF/DMSO (volume ratio, 3:1).

### Device Fabrication

The indium tin oxide (ITO) glasses were patterned by 35% hydrochloric acid and zinc powder followed by ultrasonically cleaned with acetone, and isopropyl alcohol (IPA) for at least 30 min. Then, the cleaned substrates were dried by nitrogen (N_2_) gas, and further treated by oxygen (O_2_)‐plasma at 100 W for 10 min. NiO_x_ (10 mg ml^−1^) dispersion prepared by dissolving NiO_x_ NPs power and dispersed by ultrasonication for 30 min. After the NiO_x_ dispersion was filtered by a 0.45 µm PVDF filter, NiO_x_ NPs was deposited onto the prepared substrates with a spin‐coating program at 3000 rpm for 30 s and a thermal annealing at 150 °C for 15 min. After cooling down to room temperature slowly, the substrates were transferred to the N_2_‐filled glove box. Then, 0.3 mg ml^−1^ (in ethanol) of Me‐4PACz was treated on the NiO_x_ film with a spin coating process of 3000 rpm for 25 s, and annealed at 100 °C for 10 min. Then, BDAI_2_ solution (0.5 mg ml^−1^ in IPA) was coated onto the HTL with spin coating at 4000 rpm for 30 s, and the substrate was annealed 100 °C for 10 min. Before the deposition of perovskite layer, all precursors were filtered by 0.2 µm PTFE filter. The wide‐bandgap perovskite film was spin coated at 4000 rpm for 60 s. During the spin program, 650 µl of CB was dropped at the center for the substrate after 15 s. Then, pre‐annealed at 60 °C for 2 min and further annealed at 100 °C for 10 min. After the perovskite layer deposition, 0.5 mg ml^−1^ of PDAI_2_ (in IPA) was dynamically casted with a spin rate of 4500 rpm for 30 s and annealed at 100 °C for 1 min. Twenty nanometeres of C_60_ layer was deposited by thermal evaporation system at a rate of 0.2–0.3 Å s^−1^. 0.7 mg ml^−1^ of BCP solution and 1.5 mg ml^−1^ of DTQ, DTMQ and DTMQCl solutions were prepared in IPA, and the films were spin‐coated at 4000 rpm for 30 s without annealing process. Finally, 120 nm of Ag electrode was deposited at a rate of 1.0 Å s^−1^ by thermal evaporation system, and all the thermal evaporation processes were conducted under the high vacuum condition (< 2.0 × 10^−6^ Torr). For the electron only devices, NiO_x_/Me‐4PACz layers were replaced by 30 nm of SnO_2_ layer as following structure: ITO/SnO_2_/perovskite/C_60_/interfacial layer/Ag. The SnO_2_ layer was deposited by ALD process using tetrakis(dimethylamino) tin(IV) and deionized water as precursors.

### Characterization of Organic Molecules

NMR spectra for 1H, 13C, and 31P were recorded on a JEOL JNM‐ECZ‐400 NMR spectrometer. Perkin Elmer UV–vis Lambda 365 Spectrometer was used for UV‐visible spectra measurements. Liquid chromatography mass spectrum was performed on a Bruker maXis‐HD Q‐TOF mass spectrometer. Cyclic voltammetry (CV) measurement was conducted with VersaSTAT 3 Potentiometry (Princeton Applied Research) with 0.1 m tetrabutylammonium hexafluorophosphate (TBAP) in dry acetonitrile. A glassy carbon electrode, which coated with thin film of organic materials was used as a working electrode, Pt wire was used as a counter electrode, and a reference electrode was Ag/Ag+ (0.01 m AgNO_3_). A ferrocene/ferrocenium (Fc/Fc+) redox couple was used as an external standard.

### Thin Film Characterization

Steady‐stat PL and TRPL curves were obtained using FlouTime 300 (PicoQuant). The UPS spectra were obtained using Thetaprobe (Thermo) with 21.2 eV of He I UV source. KPFM measurement was conducted at 0 V DC with a scan rate of 0.20 Hz bias using NX20 (Park Systems).

### Device Characterization

For the device characterization, the active area of the device was defined by a metal mask (0.1017 cm^2^). To obtain *J–V* curves, the measurements were performed with a scan rate of 50 mV s^−1^. The devices were illuminated under standard 100 mW cm^−2^ simulated AM 1.5G (Xenon lamp) with K3000 SS‐XE55 (McScience), calibrated by an Si photodiode (NREL‐certified) with an infrared cut‐off filter (KG5). EQE spectra were recorded with SPECTRO Mmac‐200 and ABET technology 10500 solar simulator as light solution and light source, respectively. For Mott‐Schottky, Nyquist, SCLC analysis, a potentiostat (SP‐300, BioLogic) was used, and all measurements were conducted at room temperature. The electrochemical impedance spectroscopy for Nyquist plot was obtained at 0.3 V DC bias and 0.03 V amplitude under a frequency range of 1 MHz to 1 mHz. *C–V* for Mott‐Schottky plots were performed with an AC voltage of 30 mV under a fixed frequency of 10 kHz.

## Conflict of Interest

The authors declare no conflict of interest.

## Supporting information

Supporting Information

Supplemental Data 1

Supplemental Data 2

## Data Availability

The data that support the findings of this study are available from the corresponding author upon reasonable request.
