# Supplementary material for: High Open‐Circuit Voltage Wide‐Bandgap Perovskite Solar Cell with Interface Dipole Layer
Source: Small. 2024 Aug 29;20(50):2404784. doi: 10.1002/smll.202404784 (PMC11636069; doi:10.1002/smll.202404784)
Supplement: Supplementary file 1 — Supporting Information [file SMLL-20-2404784-s003.pdf]

NANO · MICRO
small

Supporting Information

for *Small*, DOI 10.1002/smll.202404784

High Open-Circuit Voltage Wide-Bandgap Perovskite Solar Cell with Interface Dipole Layer

Jihyeon Heo, Juan Anthony Prayogo, Seok Woo Lee, Hansol Park, Senthilkumar Muthu, JeeHee Hong, Haeun Kim, Young-Hoon Kim, Dong Ryeol Whang, Dong Wook Chang and Hui Joon Park**

Supporting Information

High Open-Circuit Voltage Wide-Bandgap Perovskite Solar Cell with Interface Dipole Layer

Jihyeon Heo, Juan Anthony Prayogo, Seok Woo Lee, Hansol Park, Senthil Kumar Muthu, Jeehee Hong, Ha Eun Kim, Young-Hoon Kim, Dong Ryeol Whang, Dong Wook Chang, Hui Joon Park**

J. Heo, H. Park, S. K. Muthu, J. Hong, H. E. Kim, H. J. Park
Department of Organic and Nano Engineering
Human-Tech Convergence Program, Hanyang University, Seoul 04763, Republic of Korea
E-mail: huijoon@hanyang.ac.kr

H. J. Park
Department of Semiconductor Engineering, Hanyang University, Seoul 04763, Republic of Korea

J. A. Prayogo, S. W. Lee, D. W. Chang
Department of Industrial Chemistry and CECS Research Institute, Pukyong National University, Busan 48513, Republic of Korea
E-mail: dwchang@pknu.ac.kr

Y.-H. Kim
Department of Energy Engineering, Hanyang University, Seoul 04763, Republic of Korea

D. R. Whang
Department of Advanced Materials, Hannam University, Daejeon 34054, Republic of Korea

Keywords: perovskite solar cell, interfacial engineering, interface dipole layer, charge transport

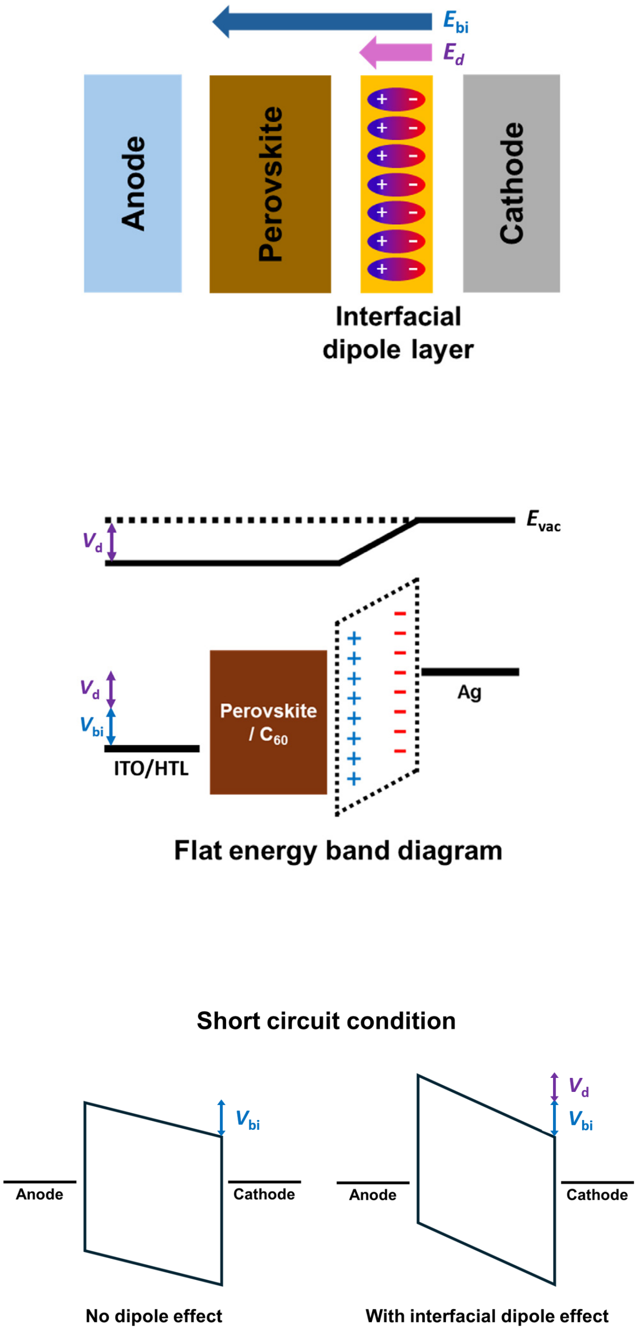

Figure S1. Energy-level diagrams of p-i-n PSC with a cathode interfacial layer composed of asymmetric molecules having intrinsic dipole moments (V_{bi} and E_{bi}: built-in potential and electric field, V_d and E_d: improved built-in potential and electric field by the dipole effect of the cathode interfacial layer).

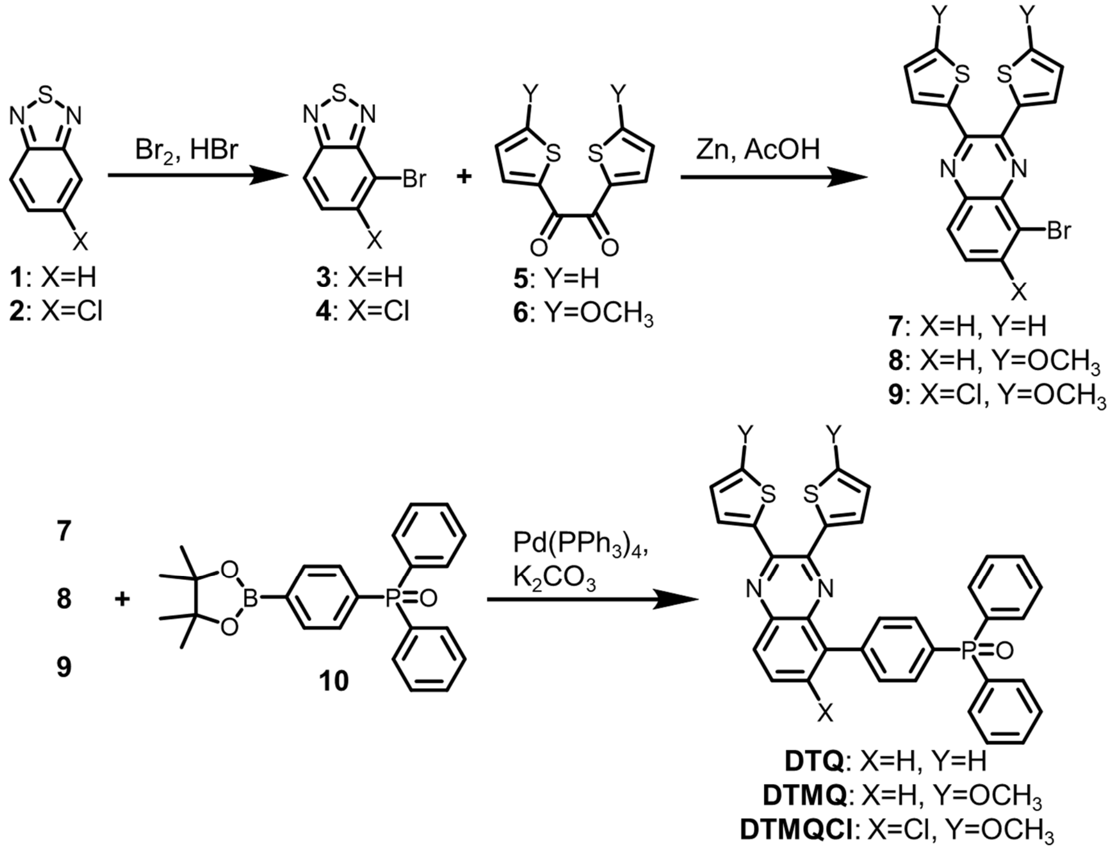

Figure S2. Synthetic routes for DTQ, DTMQ, and DTMQCl.

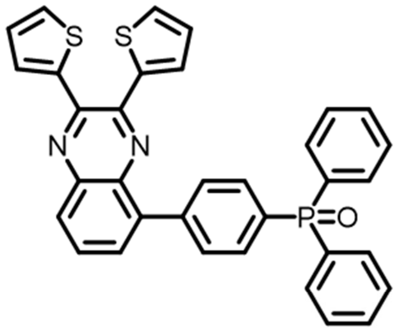

m/z: 570.0989 (100.0%), 571.1023 (36.8%), 572.0947 (9.0%), 572.1057 (3.9%), 573.0981 (3.3%), 572.1057 (2.7%), 571.0983 (1.6%)

Compound Spectrum SmartFormula Report

Analysis Info

Analysis Name	D:\Data\PKCRF\DIP\2022\0310\17_APCI_DIP_POS.d			
Method	APCI_TuneMix_50_1600mz_POS-1.m	Operator	BDAL@DE	
Sample Name	17_APCI_DIP_POS	Instrument	maxis HD	1820881.21289
Comment				

Acquisition Parameter

Source Type	APCI	Ion Polarity	Positive	Set Nebulizer	2.0 Bar
Focus	Active	Set Capillary	4500 V	Set Dry Heater	200 °C
Scan Begin	50 m/z	Set End Plate Offset	-500 V	Set Dry Gas	4.0 l/min
Scan End	1600 m/z	Set Charging Voltage	2000 V	Set Divert Valve	Waste
		Set Corona	7000 nA	Set APCI Heater	350 °C

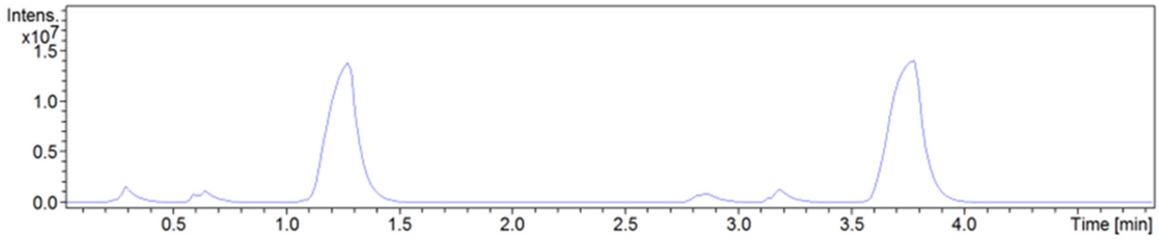

+MS, 3.6min #207

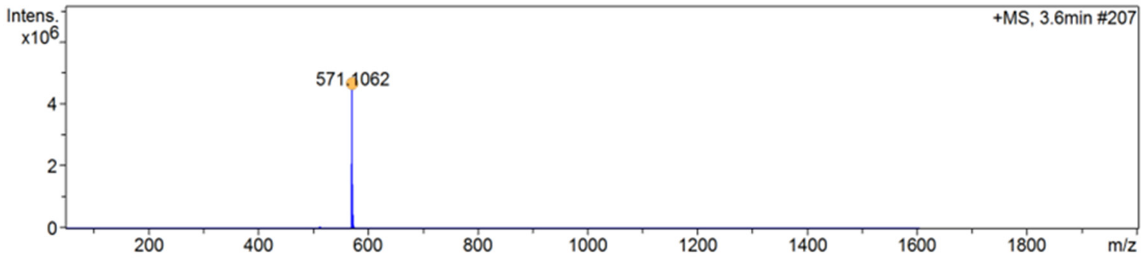

Meas. m/z	#	Ion Formula	m/z	err [ppm]	mSigma	# mSigma	Score	rdb	e⁻ Conf	N-Rule
571.1062	1	C34H24N2OPS2	571.1062	0.1	45.3	1	100.00	24.5	even	ok

Figure S3. Liquid chromatography mass spectrum of DTQ.

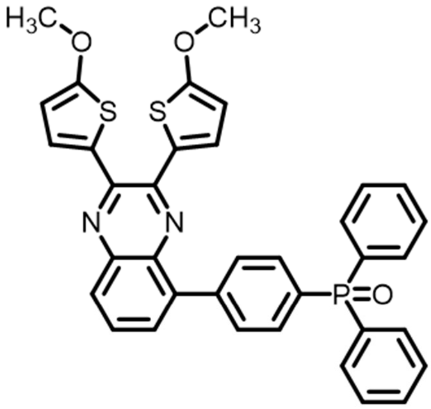

m/z: 630.1201 (100.0%), 631.1234 (38.9%), 632.1159 (9.0%)
632.1268 (7.4%), 633.1192 (3.5%), 631.1195 (1.6%)

Compound Spectrum SmartFormula Report

Analysis Info

Analysis Name	D:\Data\PKCRF\DIP\2022\0310\18_APCI_DIP_POS.d			
Method	APCI_TuneMix_50_1600mz_POS-1.m	Operator	BDAL@DE	
Sample Name	18_APCI_DIP_POS	Instrument	maxis HD	1820881.21289
Comment				

Acquisition Parameter

Source Type	APCI	Ion Polarity	Positive	Set Nebulizer	2.0 Bar
Focus	Active	Set Capillary	4500 V	Set Dry Heater	200 °C
Scan Begin	50 m/z	Set End Plate Offset	-500 V	Set Dry Gas	4.0 l/min
Scan End	1600 m/z	Set Charging Voltage	2000 V	Set Divert Valve	Waste
		Set Corona	7000 nA	Set APCI Heater	350 °C

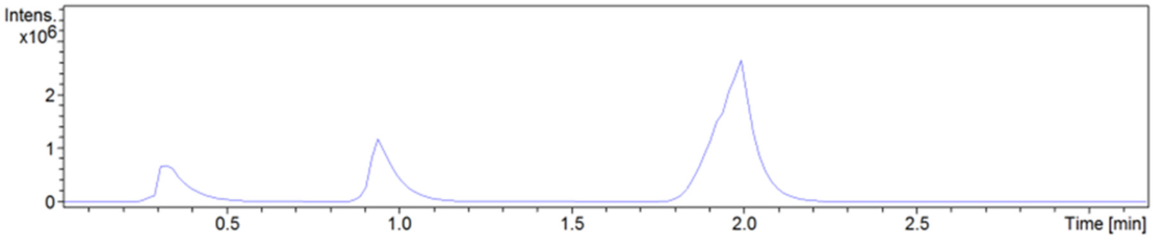

+MS, 1.9min #110

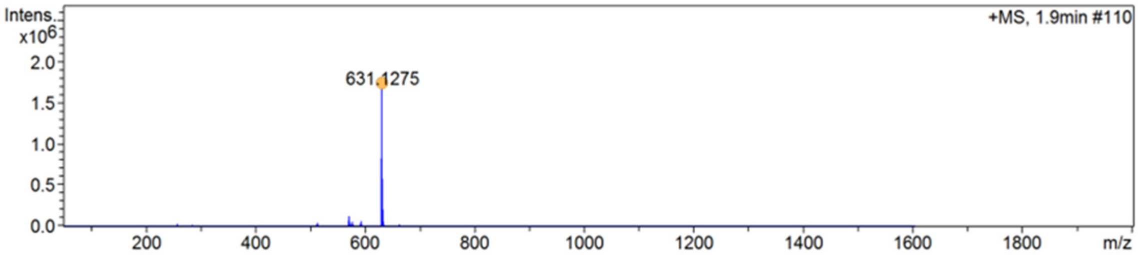

Meas. m/z	#	Ion Formula	m/z	err [ppm]	mSigma	# mSigma	Score	rdb	e⁻ Conf	N-Rule
631.1275	1	C36H28N2O3PS2	631.1273	-0.3	39.5	1	100.00	24.5	even	ok

Figure S4. Liquid chromatography mass spectrum of DTMQ.

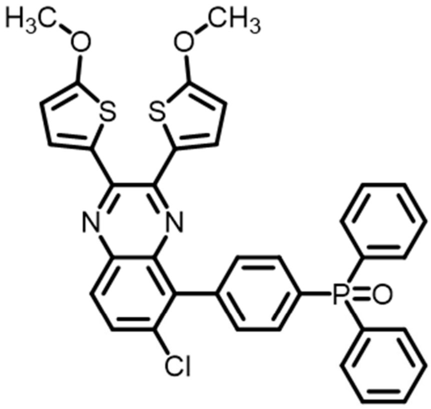

m/z: 664.0811 (100.0%), 665.0845 (38.9%), 666.0781 (32.0%), 667.0815 (12.4%), 666.0769 (9.0%), 666.0878 (4.7%), 667.0802 (3.5%), 668.0739 (2.9%), 666.0878 (2.7%), 665.0805 (1.6%), 668.0849 (1.5%), 669.0773 (1.1%)

Compound Spectrum SmartFormula Report

Analysis Info

Analysis Name	D:\Data\PKCRF\DIP\2023\0828\MTQxClTPPO_APCI_DIP_NEG.d			
Method	APCI_TuneMix_50_1600mz_Neg.m	Operator	BDAL@DE	
Sample Name	MTQxClTPPO_APCI_DIP_NEG	Instrument	maxis HD	1820881.21289
Comment				

Acquisition Parameter

Source Type	APCI	Ion Polarity	Negative	Set Nebulizer	2.5 Bar
Focus	Active	Set Capillary	4500 V	Set Dry Heater	200 °C
Scan Begin	50 m/z	Set End Plate Offset	-500 V	Set Dry Gas	2.0 l/min
Scan End	1600 m/z	Set Charging Voltage	2000 V	Set Divert Valve	Waste
		Set Corona	9000 nA	Set APCI Heater	350 °C

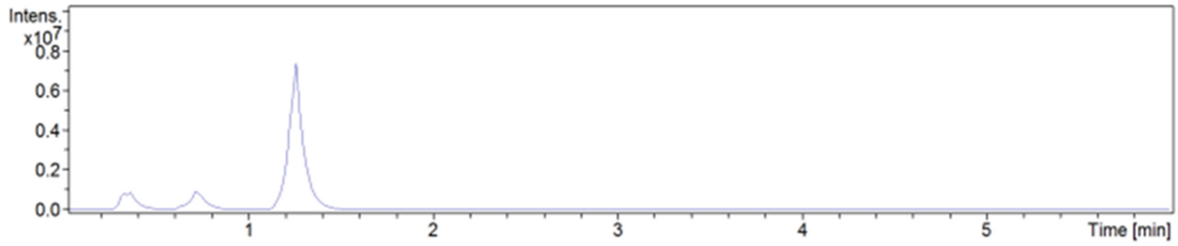

-MS, 1.2min #67

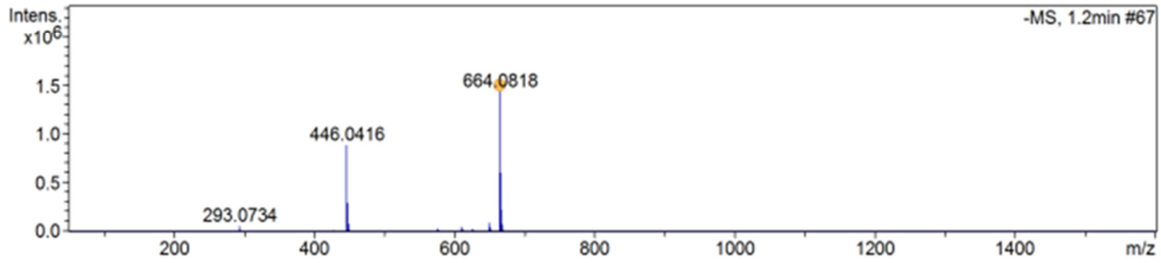

Meas. m/z	#	Ion Formula	m/z	err [ppm]	mSigma	# mSigma	Score	rdb	e⁻ Conf	N-Rule
664.0818	1	C36H26ClN2O3PS2	664.0811	-1.1	38.9	1	100.00	25.0	odd	ok

Figure S5. Liquid chromatography mass spectrum of DTMQCl.

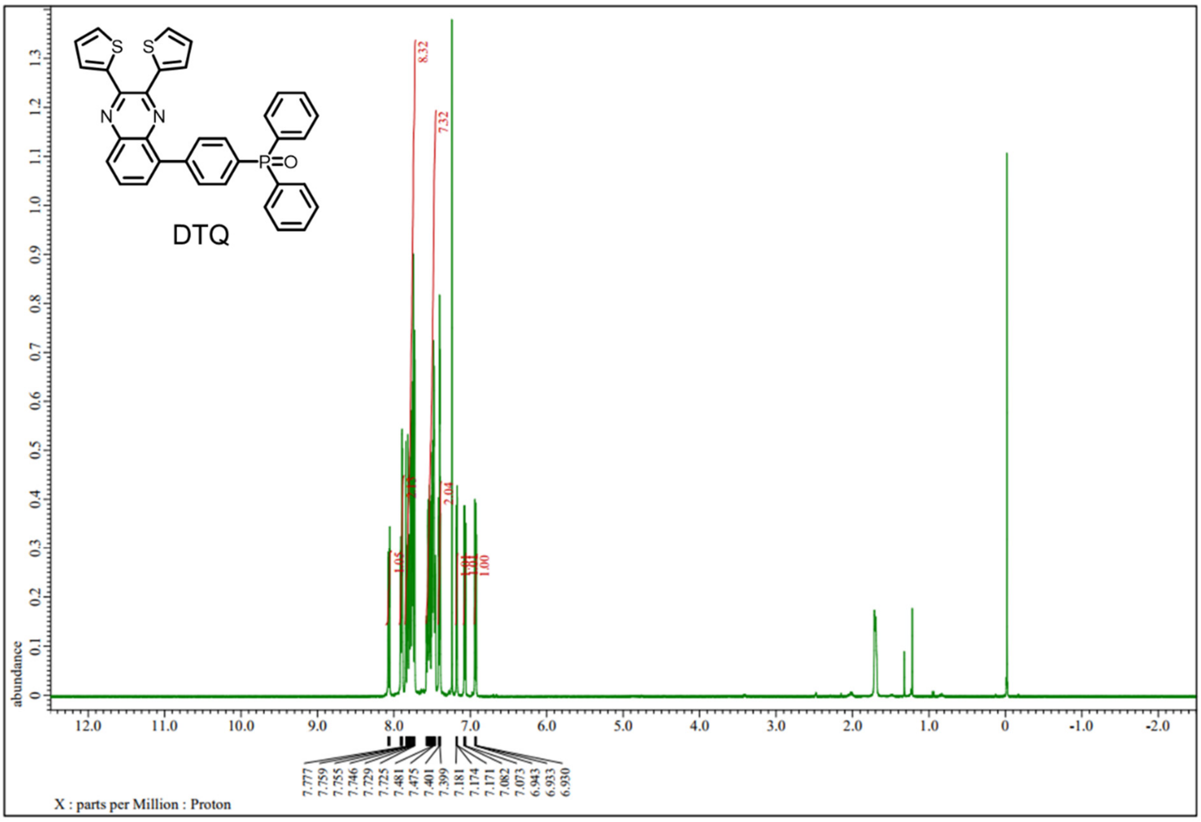

Figure S6. ^{1}H NMR (400 MHz, $CDCl_3$) δ (ppm) = 8.07 (dd, J = 8.0, 1.6 Hz, 1H), 7.89-7.92 (m, 2H), 7.73-7.85 (m, 8H), 7.46-7.57 (m, 7H), 7.40 (td, J = 4.3, 0.9 Hz, 2H), 7.18 (dd, J = 3.7, 0.9 Hz, 1H), 7.07 (dd, J = 5.0, 3.7 Hz, 1H), 6.93 (dd, J = 5.0, 3.7 Hz, 1H).

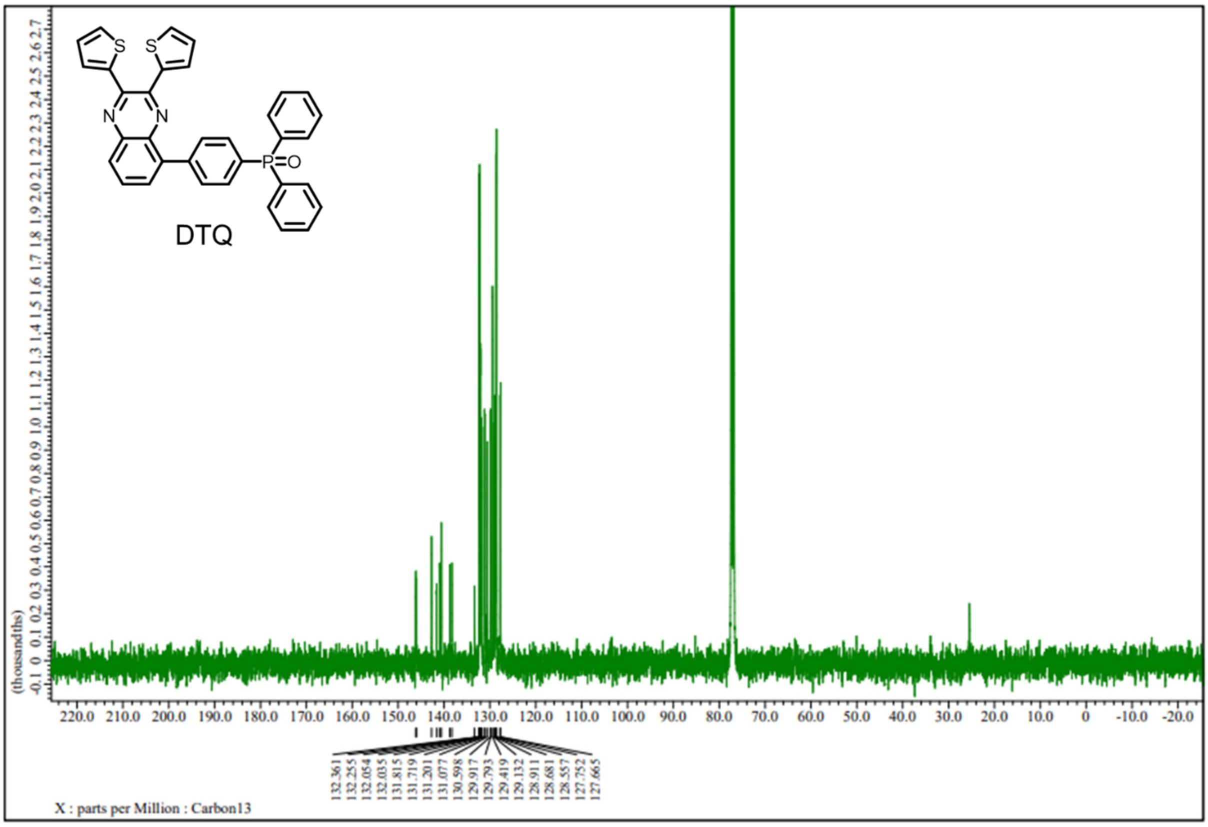

Figure S7. ^{13}C NMR (101 MHz, $CDCl_3$) δ (ppm) = 146.16, 146.00, 142.70, 141.63, 140.99, 140.55, 138.75, 138.26, 133.34, 132.36, 132.30, 132.26, 132.05, 132.04, 131.81, 131.72, 131.20, 131.08, 130.60, 129.92, 129.79, 129.42, 129.13, 128.91, 128.68, 128.56, 127.75, 127.67.

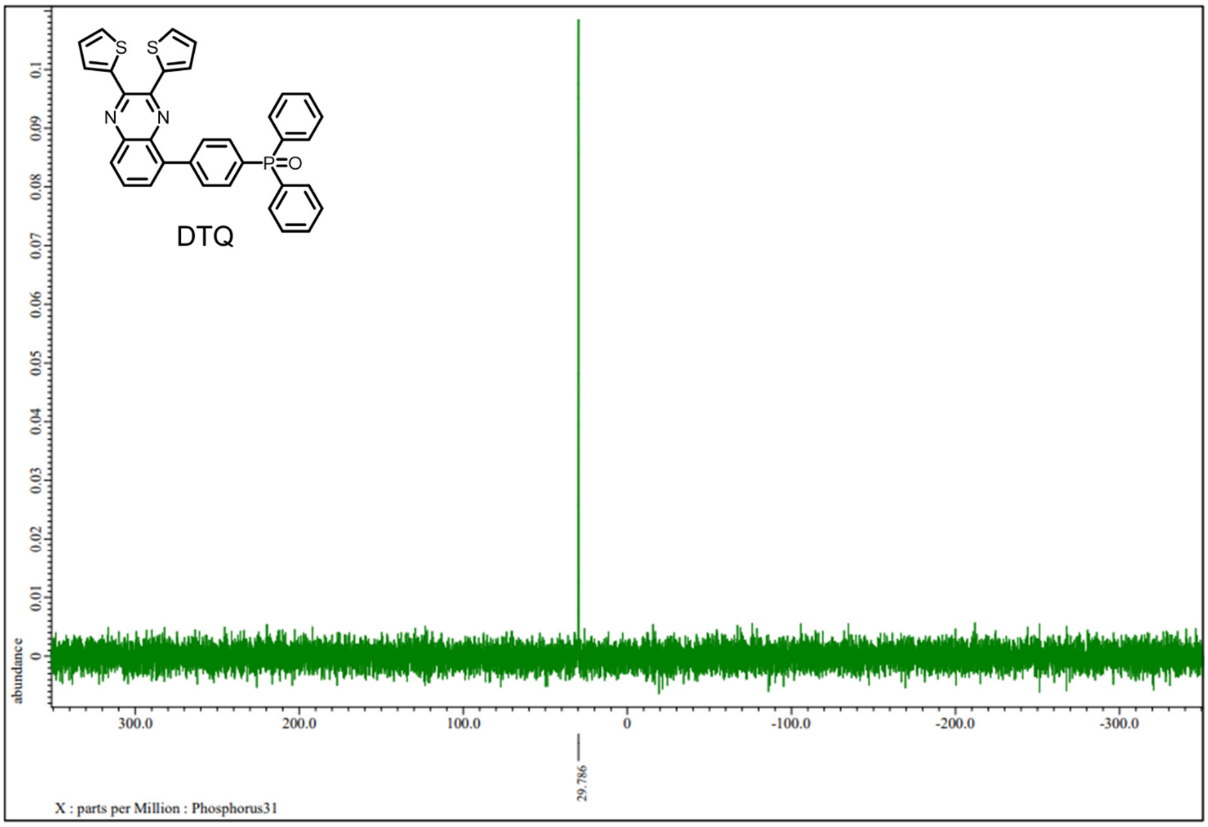

Figure S8. ^{31}P NMR (162 MHz, $CDCl_3$) δ (ppm) = 29.79.

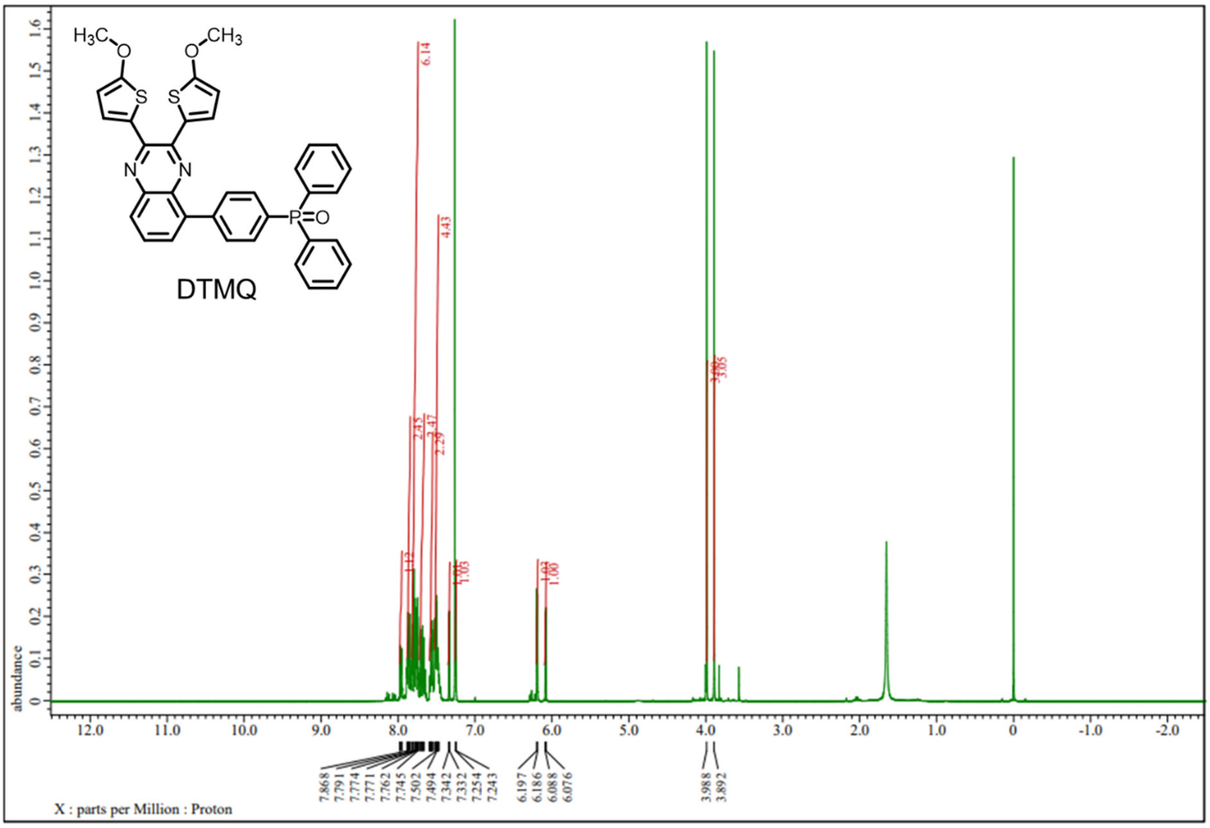

Figure S9. ^{1}H NMR (400 MHz, $CDCl_3$) δ (ppm) = 7.96 (dd, J = 8.2, 1.8 Hz, 1H), 7.84-7.88 (m, 2H), 7.74-7.82 (m, 6H), 7.66-7.72 (m, 2H), 7.55-7.59 (m, 2H), 7.47-7.51 (m, 4H), 7.34 (d, J = 4.1 Hz, 1H), 7.25 (d, J = 4.1 Hz, 1H), 6.19 (d, J = 4.1 Hz, 1H), 6.08 (d, J = 4.6 Hz, 1H), 3.99 (s, 3H), 3.89 (s, 3H).

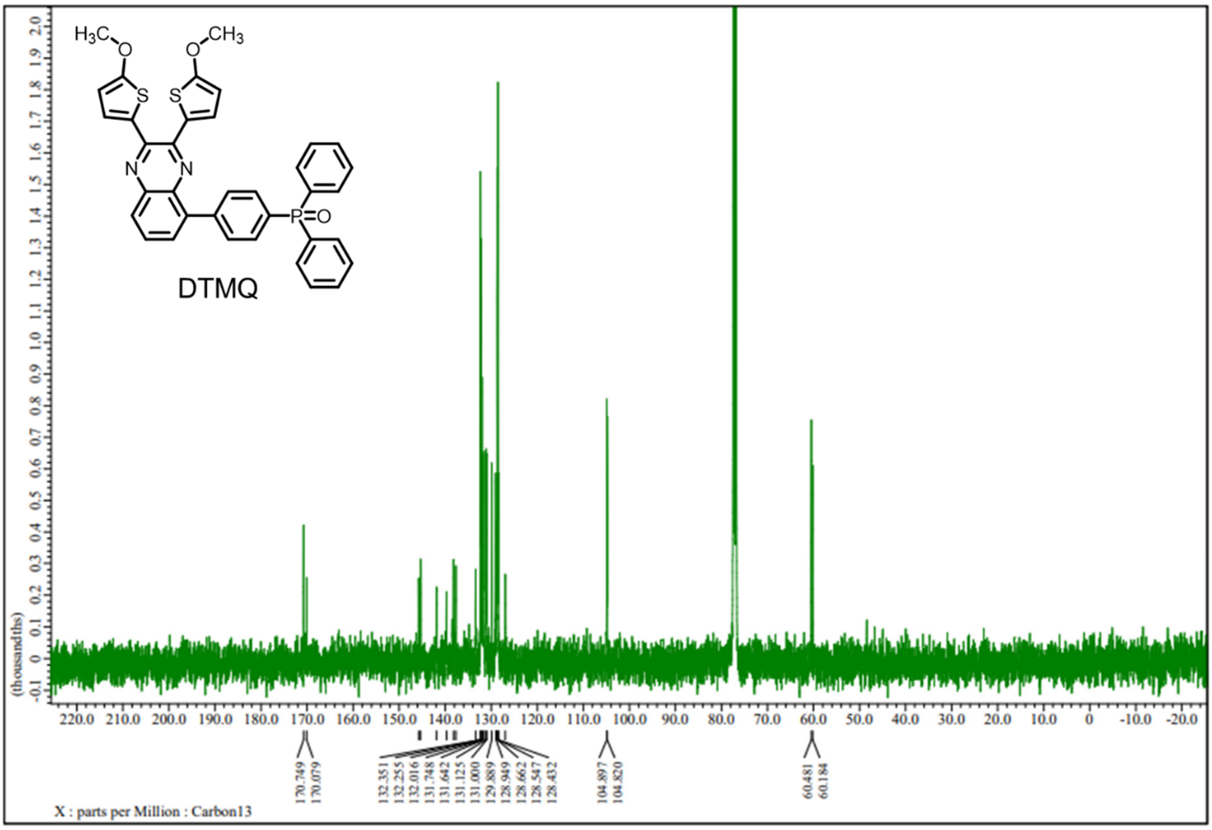

Figure S10. ^{13}C NMR (100 MHz, $CDCl_3$) δ (ppm) = 170.75, 170.08, 145.76, 145.31, 141.85, 139.71, 138.21, 137.67, 133.40, 132.35, 132.26, 132.02, 131.75, 131.64, 131.12, 131.00, 129.89, 128.99, 128.95, 128.66, 128.55, 128.43, 126.96, 104.90, 104.82, 60.48, 60.18.

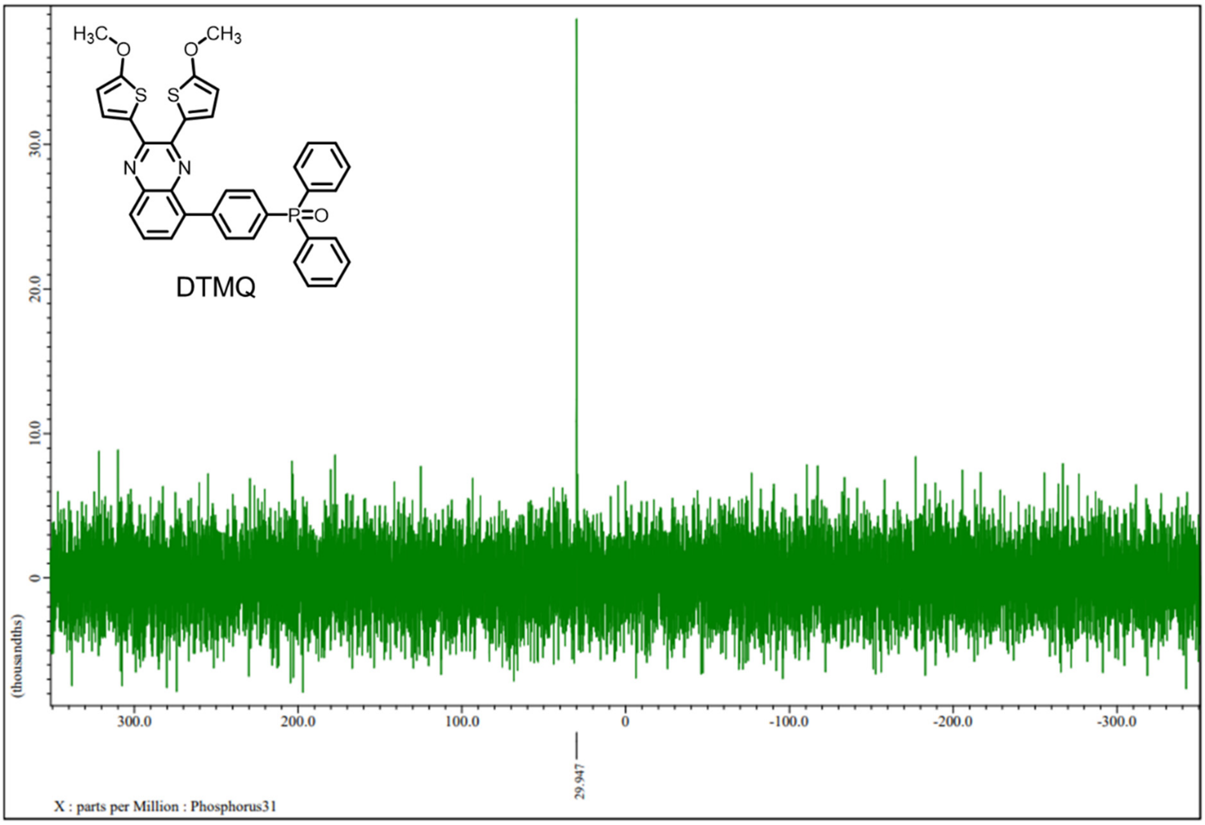

Figure S11. ^{31}P NMR (162 MHz, $CDCl_3$) δ (ppm) = 29.95.

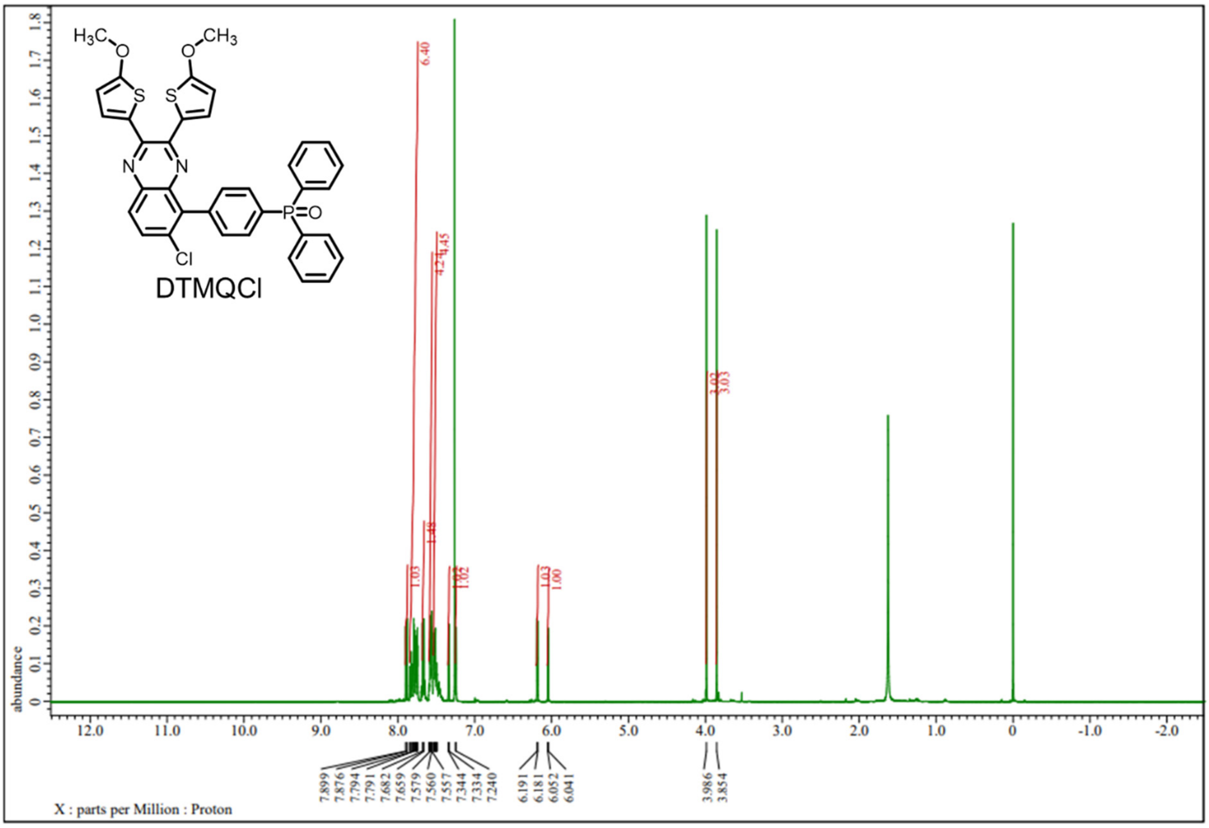

Figure S12. ^{1}H NMR (400 MHz, $CDCl_3$) δ (ppm) = 7.89 (d, J = 9.1 Hz, 1H), 7.74-7.84 (m, 6H), 7.67 (d, J = 9.1 Hz, 1H), 7.56-7.60 (m, 4H), 7.49-7.54 (m, 4H), 7.34 (d, J = 4.1 Hz, 1H), 7.25 (d, J = 4.1 Hz, 1H), 6.19 (d, J = 4.1 Hz, 1H), 6.05 (d, J = 4.6 Hz, 1H), 3.99 (s, 3H), 3.85 (s, 3H).

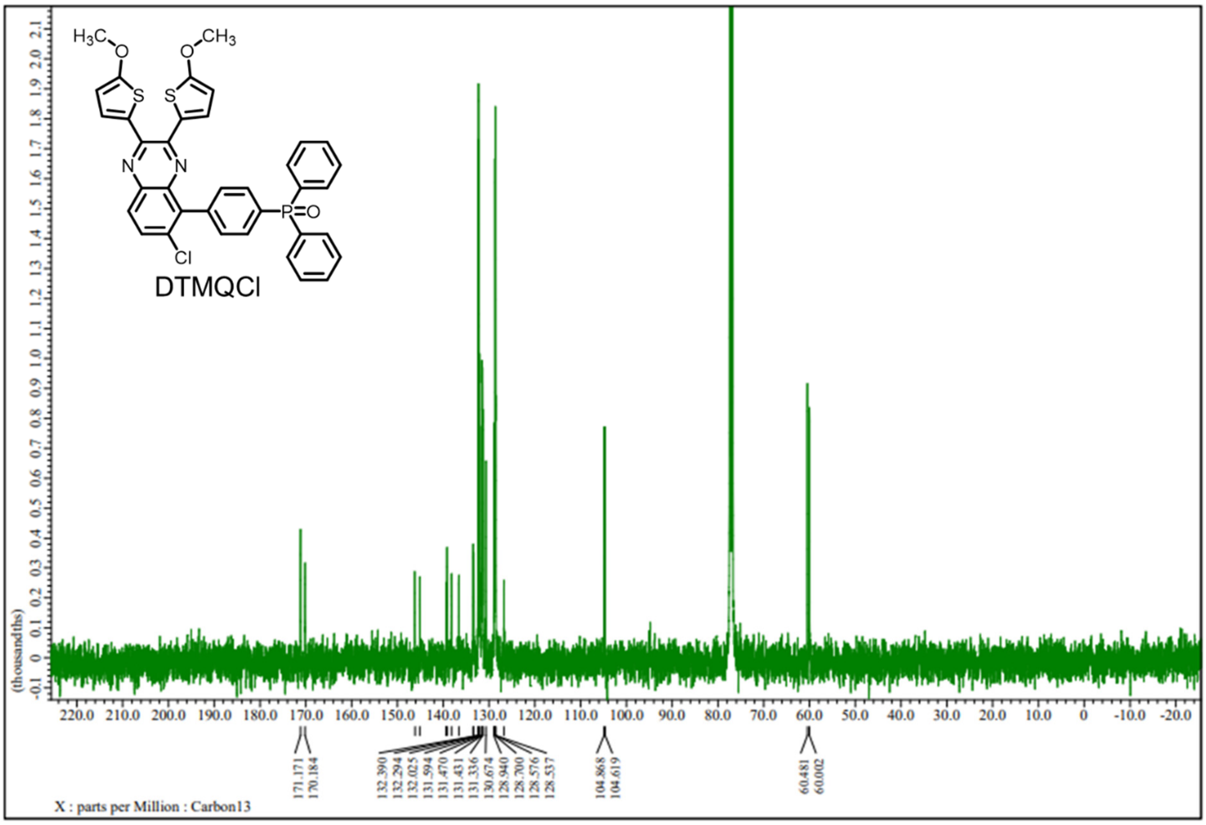

Figure S13. ^{13}C NMR (100 MHz, $CDCl_3$) δ (ppm) = 171.17, 170.18, 146.12, 145.12, 139.35, 139.32, 139.18, 138.18, 136.59, 133.47, 133.34, 132.39, 132.29, 132.03, 131.59, 131.47, 131.43, 131.34, 130.67, 128.94, 128.81, 128.70, 128.58, 128.54, 126.73, 104.87, 104.62, 60.48, 60.00.

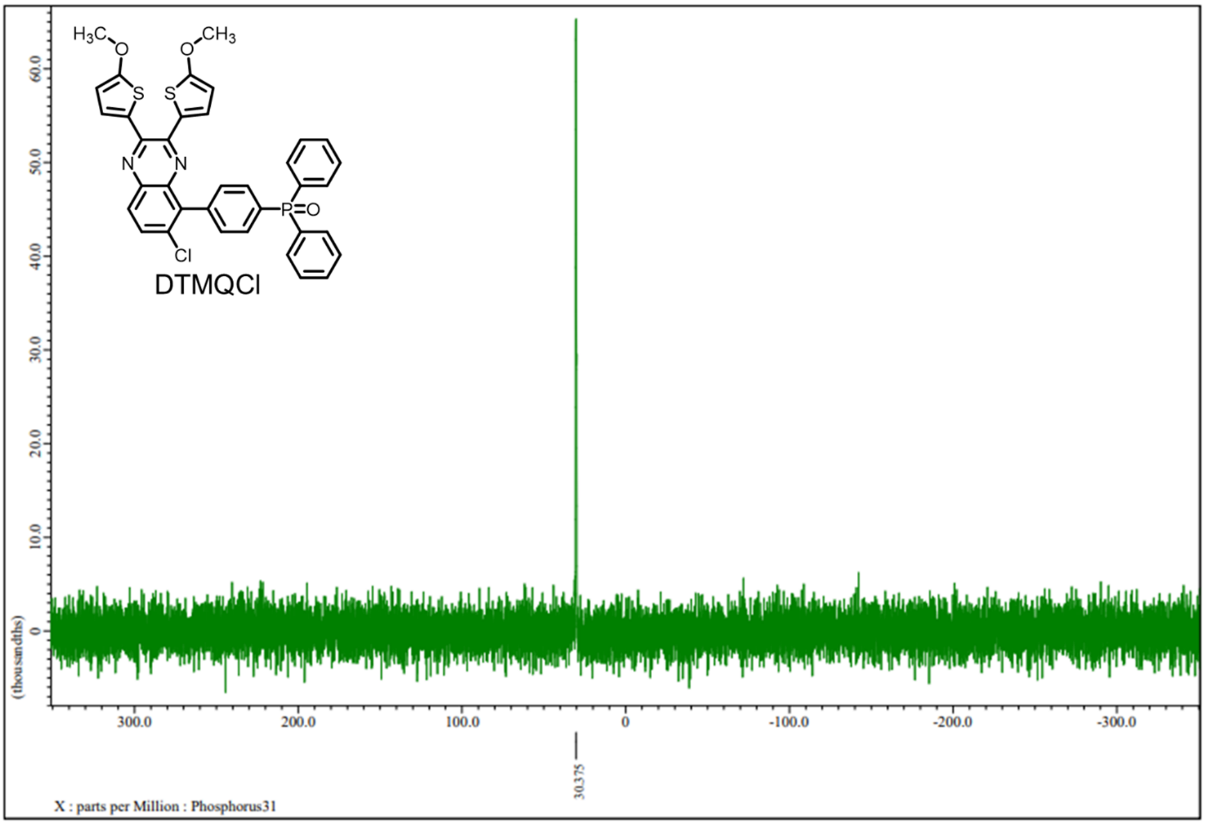

Figure S14. ^{31}P NMR (162 MHz, $CDCl_3$) δ (ppm) = 30.38.

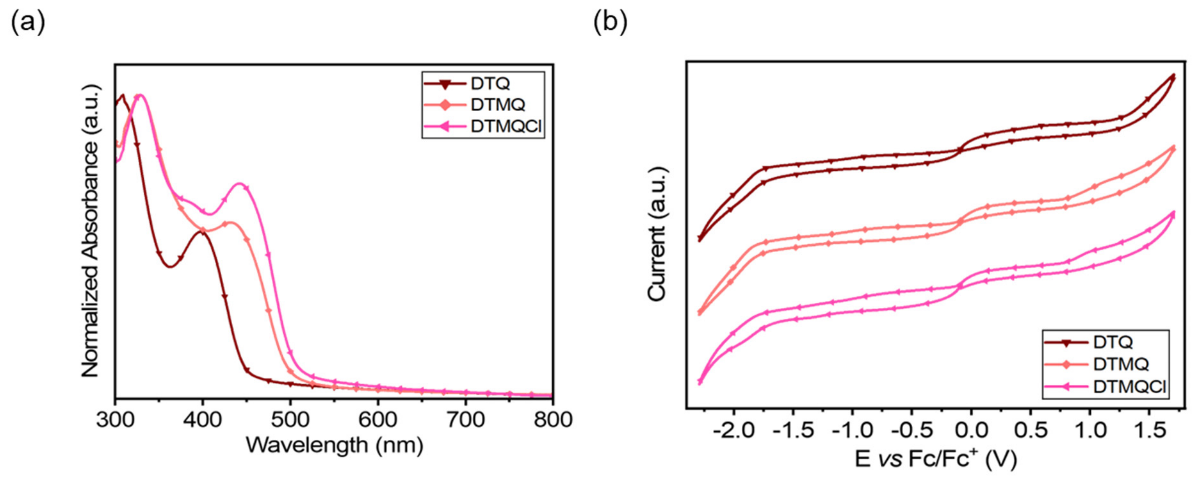

Figure S15. (a) UV-vis spectra of DTQ, DTMQ, and DTMQCl in films. (b) CV curves of DTQ, DTMQ, and DTMQCl.

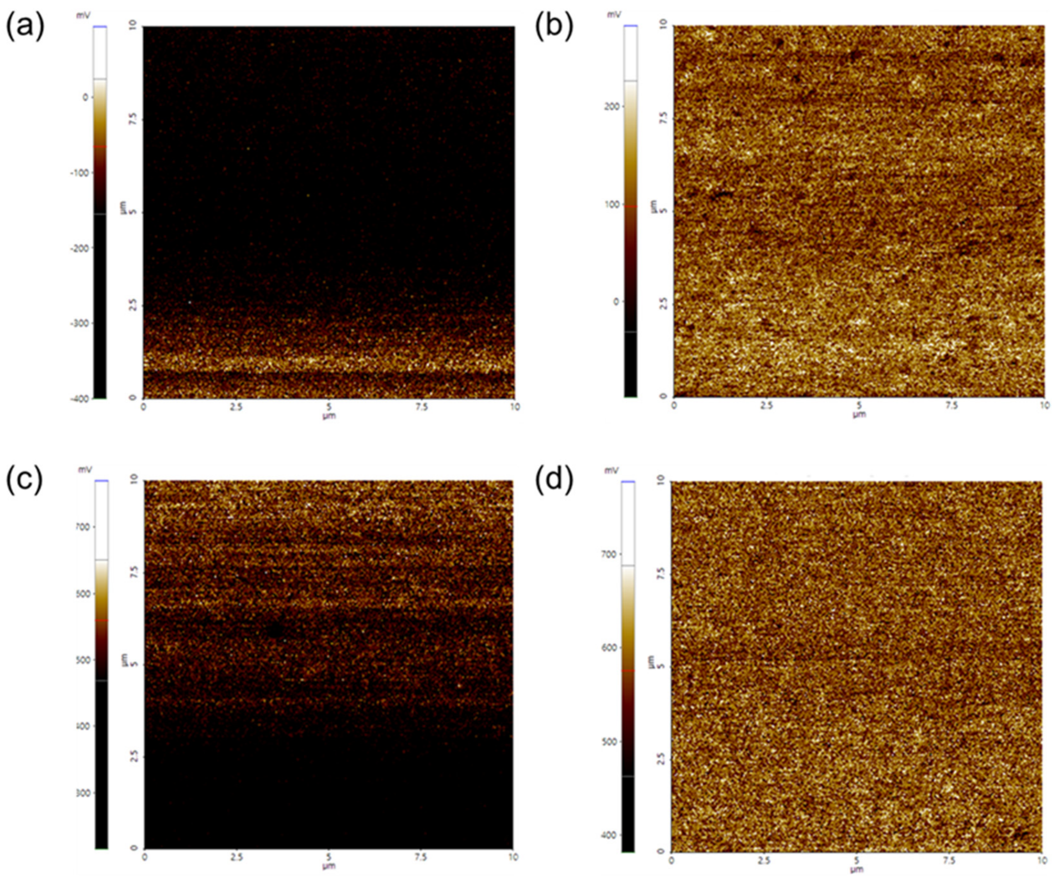

Figure S16. Surface potential maps from KPFM measurement for (a) Ag without organic interfacial layer on (b) DTQ, (c) DTMQ, and (d) DTMQCl.

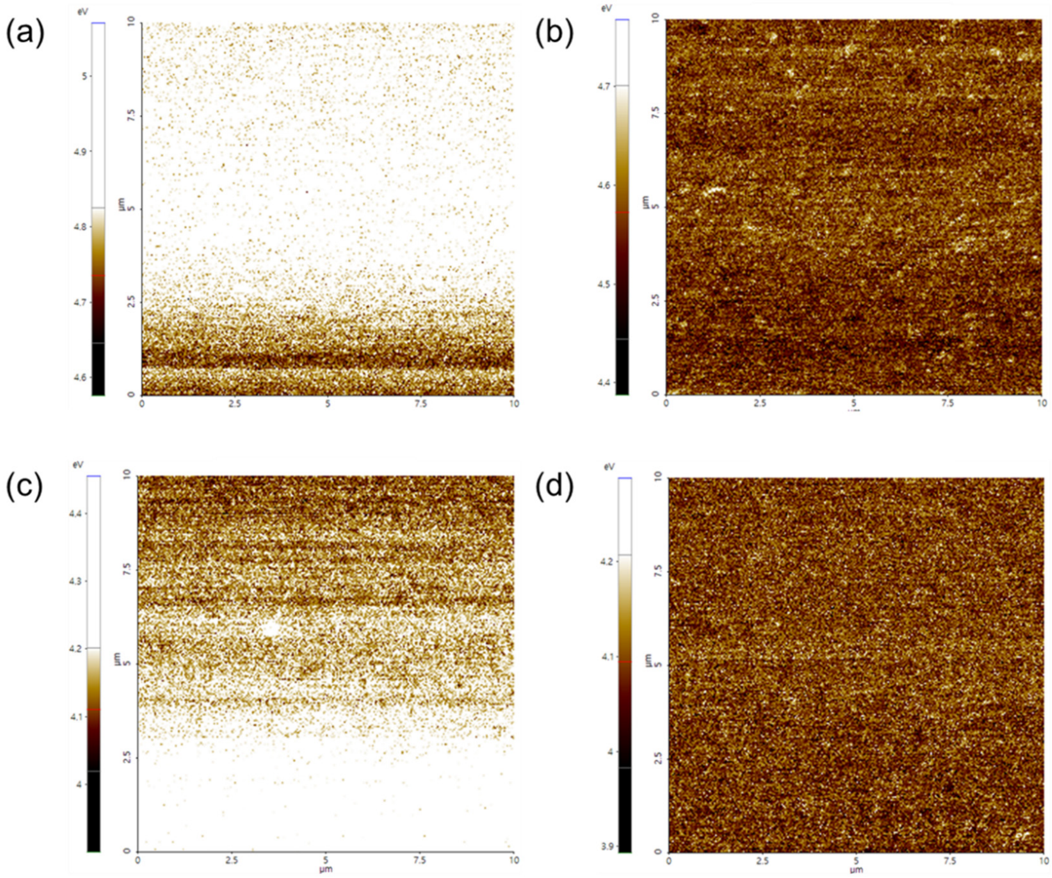

Figure S17. Estimated work function from KPFM measurement for (a) Ag without organic interfacial layer and Ag layers on (b) DTQ, (c) DTMQ, and (d) DTMQCl.

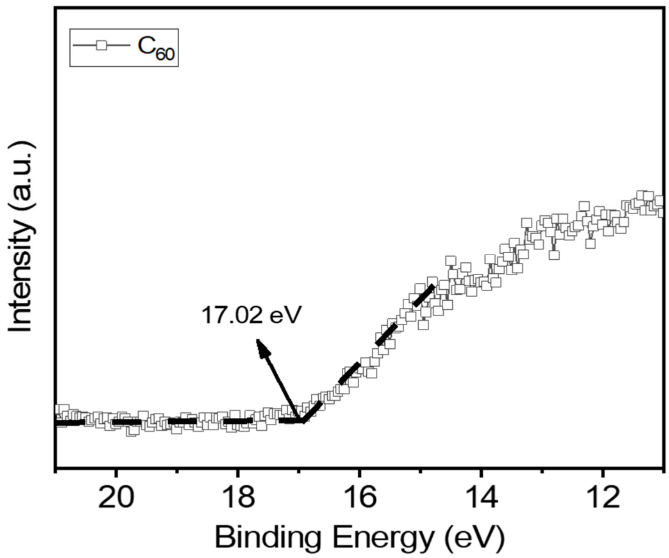

Figure S18. Ultraviolet photoelectron spectroscopy (UPS) binding energy profile representing the secondary electron cut-off energy associated with the work functions of C_{60}.

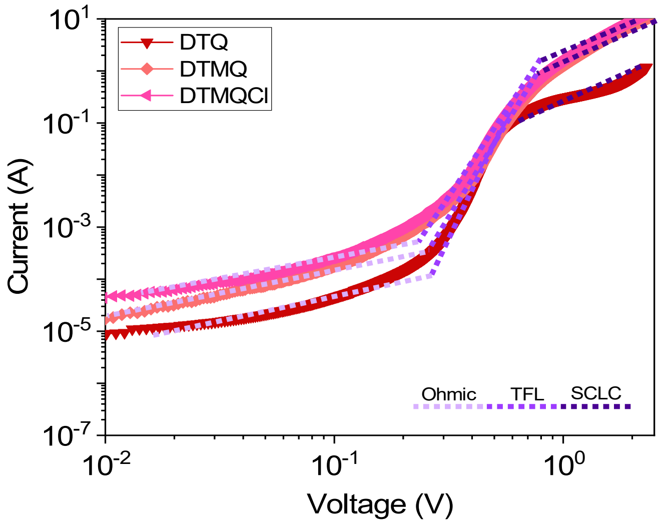

Figure S19. Dark *J-V* curves of the electron-only devices (ITO/SnO_2/perovskite/C_{60}/interfacial layer/Ag) with DTQ, DTMQ, and DTMQCl.

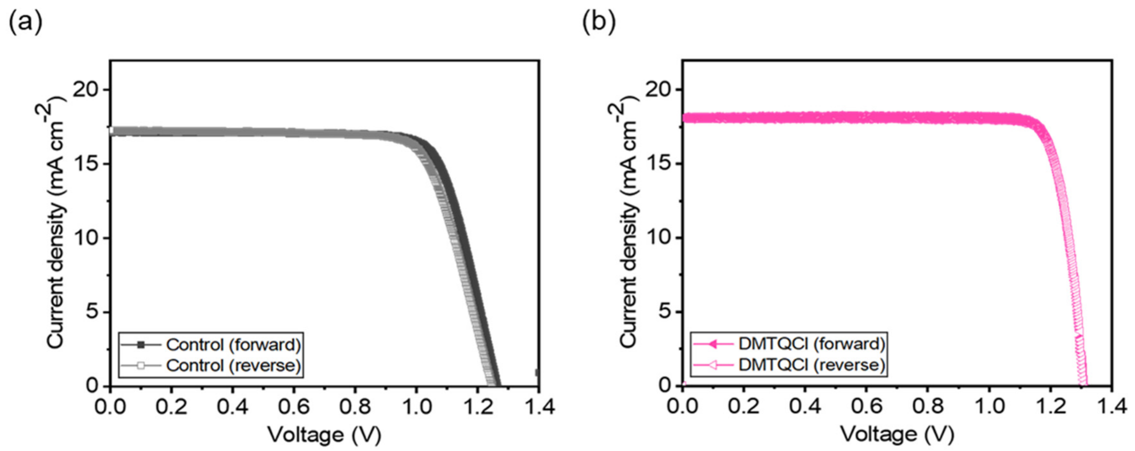

Figure S20. *J-V* curves in forward and reverse scans of the PSC (simulated AM 1.5G at 100 mW cm^{-2}) (a) without interfacial layer and (b) with DTMQCl interfacial layer.

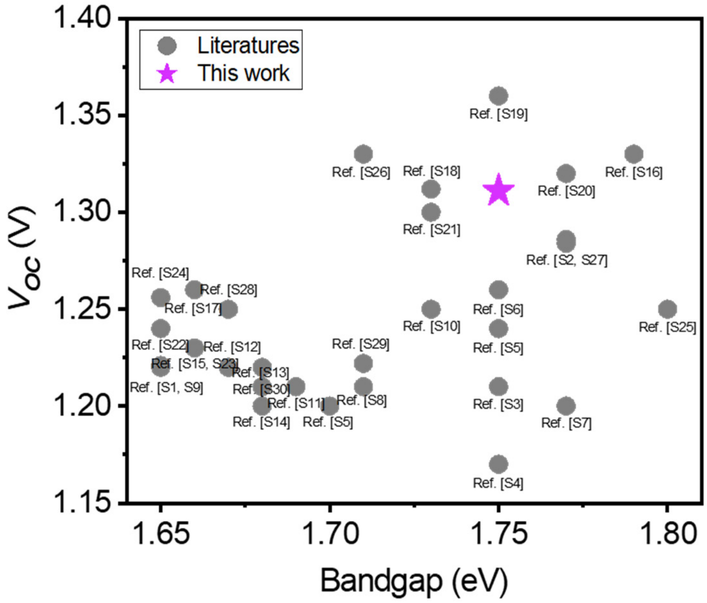

Figure S21. Open-circuit voltage (V_{oc}) values of PSCs as a function of bandgap from the literature, including this work.

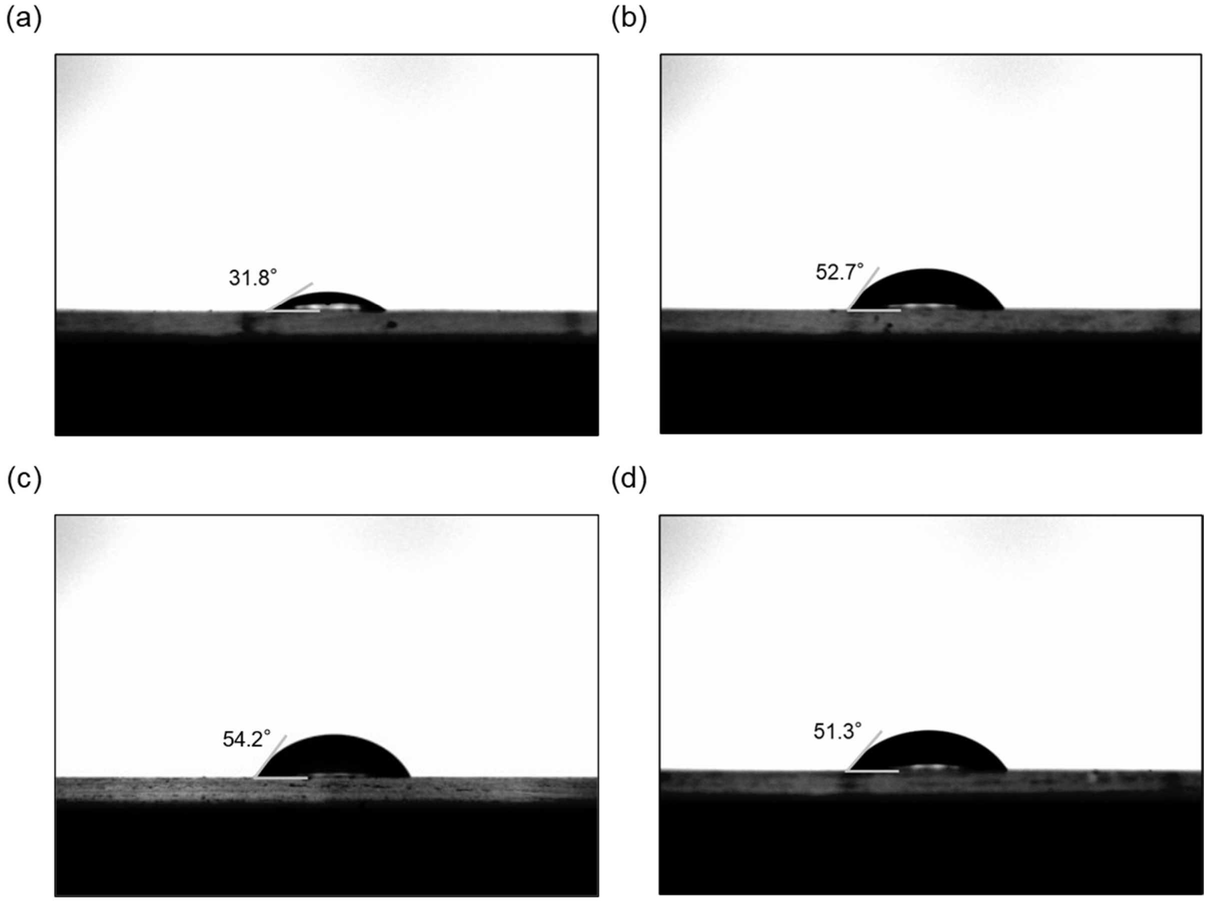

Figure S22. Contact angles of (a) C_{60}, (b) DTQ, (c)DTMQ and (b) DTMQCl films.

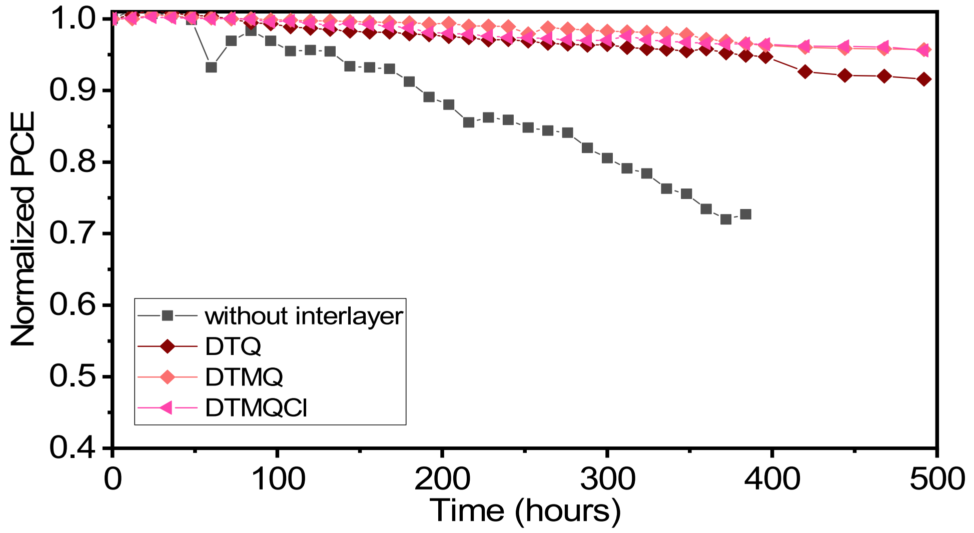

Figure S23. Stability of PSC devices without and with organic interfacial layers (DTQ, DTMQ, and DTMQCl) under ambient condition with a relative humidity of 30-50%.

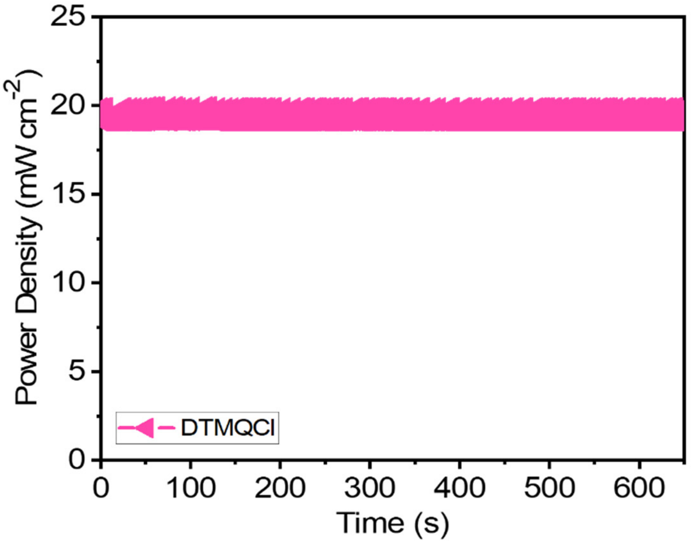

Figure S24. Maximum power point tracking (MPPT) result of the device with DTMQCl.

Table S1. PL decay lifetimes of perovskite films with and without C_{60} ETL and organic interfacial layers, fitted by a biexponential equation: $\tau_{avg} = A_1 \exp\left(-\frac{t}{\tau_1}\right) + A_2 \exp\left(-\frac{t}{\tau_2}\right)$

Structure	A_1 (%)	τ_1 (ns)	A_2 (%)	τ_2 (ns)	τ_{avg} (ns)
**Perovskite**	30.45	7.14	63.10	129.87	84.12
**Perovskite/C_{60}**	60.65	1.50	38.15	14.47	6.43
**Perovskite/C_{60}/BCP**	66.26	2.20	33.62	11.10	5.19
**Perovskite/C_{60}/DTQ**	70.42	2.54	28.51	10.29	4.71
**Perovskite/C_{60}/DTMQ**	54.60	1.95	45.11	8.02	4.68
**Perovskite/C_{60}/DTMQCl**	60.48	1.28	39.31	3.97	2.34

Table S2. Series (R_s) and recombination resistance (R_{rec}) values of PSC devices with and without interfacial layers, estimated by Nyquist plots from electrochemical impedance spectroscopy (EIS).

**Structure**	R_s (Ω)	R_{rec} (Ω)
**without interfacial layer**	14.1	975.4
**BCP**	8.1	1192.2
**DTQ**	9.5	1719.7
**DTMQ**	7.6	1973.4
**DTMQCl**	7.4	2463.4

Table S3. Photovoltaic parameters of PSC devices with and without interfacial layer (DTMQCl) depending on the scan direction: forward scan (FS) and reverse scan (RS).

	V_{oc} (V)	J_{sc} (mA/cm^2)	Fill Factor	PCE (%)	HI
**without interfacial layer (FS)**	1.26	17.2	76.8	16.7	-0.05
**without interfacial layer (RS)**	1.25	17.0	74.9	15.9	
**DTMQCl (FS)**	1.31	18.1	85.6	20.3	-0.01
**DTMQCl (RS)**	1.31	18.1	85.1	20.1	

Table S4. Photovoltaic parameters of wide-bandgap PSCs in literature.

Ref	Perovskite	Bandgap (eV)	V_{oc} (V)	V_{oc} loss (V)	PCE (%)
This work	$Cs_{0.16}FA_{0.80}MA_{0.04}Pb(I_{0.68}Br_{0.32})_3$	1.75	1.31	0.44	20.3
[S1]	$FA_{0.8}Cs_{0.15}MA_{0.05}Pb(I_{0.82}Br_{0.18})_3$	1.65	1.221	0.429	21.9
[S2]	$FA_{0.8}Cs_{0.2}Pb(I_{0.6}Br_{0.4})_3$	1.77	1.286	0.484	18.63
[S3]	$MAPb(I_{0.64}Br_{0.26})_3$	1.75	1.21	0.54	14.9
[S4]	$FA_{0.75}Cs_{0.25}Pb(I_{0.8}Br_{0.2})_3$	1.75	1.17	0.58	16.3
[S5]	$FA_{0.8}Cs_{0.2}Pb(I_{0.7}Br_{0.3})_3$	1.75	1.24	0.51	18.19
	$DMA_{0.1}Cs_{0.3}FA_{0.6}PbI_{2.4}Br_{0.6}$	1.7	1.2	0.5	19.2
[S6]	$(FA_{0.6}MA_{0.4})_{0.9}Cs_{0.1}Pb(I_{0.6}Br_{0.4})_3$	1.75	1.26	0.49	18.3
[S7]	$FA_{0.8}Cs_{0.2}Pb(I_{0.6}Br_{0.4})_3$	1.77	1.2	0.57	16.4
[S8]	$Cs_{0.05}(FA_{0.83}MA_{0.17})_{0.95}Pb(I_{0.6}Br_{0.4})_3$	1.71	1.21	0.5	18.5
[S9]	$Cs_{0.05}FA_{0.8}MA_{0.15}Pb(I_{0.75}Br_{0.25})_3$	1.65	1.22	0.43	20.7
[S10]	$Cs_{0.2}FA_{0.8}Pb(I_{0.7}Br_{0.3})_3$	1.73	1.25	0.48	19.07
[S11]	$Cs_{0.05}(FA_{0.76}MA_{0.24})_{0.95}Pb(I_{0.76}Br_{0.24})_3$	1.69	1.21	0.48	20.1
[S12]	$Cs_{0.25}FA_{0.75}Pb(I_{0.8}Br_{0.15}Cl_{0.05})_3$	1.67	1.22	0.45	20.42
[S13]	$Cs_{0.15}FA_{0.70}MA_{0.15}Pb(I_{0.80}Br_{0.20})_3$	1.68	1.22	0.46	20.5
[S14]	$Cs_{0.22}FA_{0.78}PbI_{2.55-x}Br_{0.45}Cl_x$	1.68	1.2	0.48	20.39
[S15]	$Cs_{0.2}FA_{0.8}Pb(I_{0.82}Br_{0.15}Cl_{0.03})_3$	1.66	1.23	0.43	21.05
[S16]	$Cs_{0.2}FA_{0.8}Pb(I_{0.60}Br_{0.40})_3$	1.79	1.33	0.46	20.2
[S17]	$FA_{0.75}Cs_{0.25}Pb(I_{0.8}Br_{0.2})_3$	1.66	1.26	0.4	22.4
[S18]	$FA_{0.8}Cs_{0.2}Pb(I_{0.7}Br_{0.3})_3$	1.73	1.312	0.418	20.22
[S19]	$FA_{0.8}Cs_{0.2}Pb(I_{0.6}Br_{0.4})_3$	1.75	1.36	0.39	19.83
[S20]	$FA_{0.6}Cs_{0.4}Pb(I_{0.7}Br_{0.3})_3$	1.77	1.32	0.45	19.31
[S21]	$FA_{0.8}MA_{0.1}Cs_{0.1}Pb(I_{0.7}Br_{0.3})_3$	1.73	1.3	0.43	21.3
[S22]	$[Cs_{0.22}FA_{0.78}Pb(I_{0.85}Br_{0.15})_3]_{0.97}(MAPbCl_3)_{0.03}$	1.65	1.24	0.41	21.55
[S23]	$FA_{0.75}Cs_{0.25}Pb(I_{0.8}Br_{0.2})_3$	1.66	1.23	0.43	21.47

[S24]	$FA_{0.8}Cs_{0.15}MA_{0.05}PbI_{2.4}Br_{0.6}$	1.65	1.256	0.394	21.9
[S25]	$FA_{0.83}Cs_{0.17}Pb(I_{0.6}Br_{0.4})_3$	1.8	1.25	0.55	17.4
[S26]	$CsPbI_3$	1.71	1.33	0.38	17
[S27]	$FA_{0.8}Cs_{0.2}Pb(I_{0.6}Br_{0.4})_3$	1.77	1.284	0.486	17.72
[S28]	$FA_{0.7}Cs_{0.25}MA_{0.05}Pb(I_{0.8}Br_{0.2})_3$	1.67	1.25	0.42	22.06
[S29]	$CsPbI_{2.85}Br_{0.15}$	1.71	1.222	0.488	20.38
[S30]	$Rb_{0.05}Cs_{0.05}(FA_{0.75}MA_{0.25})Pb(I_{0.75}Br_{0.25})_3$	1.68	1.21	0.47	20.11

Supplementary references:

[S1] Z. Liu, C. Zhu, H. Luo, W. Kong, X. Luo, J. Wu, C. Ding, Y. Chen, Y. Wang, J. Wen, Y. Gao, H. Tan, *Adv. Energy Mater.* **2023**, *13*, 2203230.

[S2] W. Wang, X. Liu, J. Wang, C. Chen, J. Yu, D. Zhao, W. Tang, *Adv. Energy Mater.* **2023**, *13*, 2300694.

[S3] M. Hu, C. Bi, Y. Yuan, Y. Bai, J. Huang, *Adv. Sci.* **2016**, *3*, 1500301.

[S4] K. A. Bush, K. Frohna, R. Prasanna, R. E. Beal, T. Leijtens, S. A. Swifter, M. D. McGehee, *ACS Energy Lett.* **2018**, *3*, 428.

[S5] C. Chen, Z. Song, C. Xiao, D. Zhao, N. Shrestha, C. Li, G. Yang, F. Yao, X. Zheng, R. J. Ellingson, *Nano Energy* **2019**, *61*, 141.

[S6] Z. Li, J. Zhang, S. Wu, X. Deng, F. Li, D. Liu, C. C. Lee, F. Lin, D. Lei, C.-C. Chueh, Z. Zhu, A. K. Y. Jen, *Nano Energy* **2020**, *78*, 105377.

[S7] K. Xiao, R. Lin, Q. Han, Y. Hou, Z. Qin, H. T. Nguyen, J. Wen, M. Wei, V. Yeddu, M. I. Saidaminov, Y. Gao, X. Luo, Y. Wang, H. Gao, C. Zhang, J. Xu, J. Zhu, E. H. Sargent, H. Tan, *Nat. Energy* **2020**, *5*, 870.

[S8] Y. Lin, B. Chen, F. Zhao, X. Zheng, Y. Deng, Y. Shao, Y. Fang, Y. Bai, C. Wang, J. Huang, *Adv. Mater.* **2017**, *29*, 1700607.

[S9] H. Tan, F. Che, M. Wei, Y. Zhao, M. I. Saidaminov, P. Todorović, D. Broberg, G. Walters, F. Tan, T. Zhuang, B. Sun, Z. Liang, H. Yuan, E. Fron, J. Kim, Z. Yang, O. Voznyy, M. Asta, E. H. Sargent, *Nat. Commun.* **2018,** *9*, 3100.

[S10] C. Chen, Z. Song, C. Xiao, R. A. Awni, C. Yao, N. Shrestha, C. Li, S. S. Bista, Y. Zhang, L. Chen, R. J. Ellingson, C.-S. Jiang, M. Al-Jassim, G. Fang, Y. Yan, *ACS Energy Lett.* **2020**, *5*, 2560.

[S11] F. Peña-Camargo, P. Caprioglio, F. Zu, E. Gutierrez-Partida, C. M. Wolff, K. Brinkmann, S. Albrecht, T. Riedl, N. Koch, D. Neher, M. Stolterfoht, *ACS Energy Lett.* **2020**, *5*, 2728.

[S12] J. Xu, C. C. Boyd, J. Y. Zhengshan, A. F. Palmstrom, D. J. Witter, B. W. Larson, R. M. France, J. Werner, S. P. Harvey, E. J. Wolf, *Science* **2020**, *367*, 1097.

[S13] F. H. Isikgor, F. Furlan, J. Liu, E. Ugur, M. K. Eswaran, A. S. Subbiah, E. Yengel, M. De Bastiani, G. T. Harrison, S. Zhumagali, C. T. Howells, E. Aydin, M. Wang, N.

Gasparini, T. G. Allen, A. ur Rehman, E. Van Kerschaver, D. Baran, I. McCulloch, T. D. Anthopoulos, U. Schwingenschlögl, F. Laquai, S. De Wolf, *Joule* **2021**, *5*, 1566.

[S14] Z. M. Fang, L. B. Jia, N. Yan, X. F. Jiang, X. D. Ren, S. F. Yang, S. Z. Liu, *InfoMat* **2022**, *4*, e12307.

[S15] D. Wang, H. Guo, X. Wu, X. Deng, F. Li, Z. Li, F. Lin, Z. Zhu, Y. Zhang, B. Xu, A. K. Y. Jen, *Adv. Funct. Mater.* **2021**, *32*, 2107359.

[S16] H. Chen, A. Maxwell, C. Li, S. Teale, B. Chen, T. Zhu, E. Ugur, G. Harrison, L. Grater, J. Wang, Z. Wang, L. Zeng, S. M. Park, L. Chen, P. Serles, R. A. Awni, B. Subedi, X. Zheng, C. Xiao, N. J. Podraza, T. Filleter, C. Liu, Y. Yang, J. M. Luther, S. De Wolf, M. G. Kanatzidis, Y. Yan, E. H. Sargent, *Nature* **2023**, *613*, 676.

[S17] H. Guan, S. Zhou, S. Fu, D. Pu, X. Chen, Y. Ge, S. Wang, C. Wang, H. Cui, J. Liang, X. Hu, W. Meng, G. Fang, W. Ke, *Adv. Mater.* **2024**, *36*, 2307987.

[S18] Y. Zhao, C. Wang, T. Ma, L. Zhou, Z. Wu, H. Wang, C. Chen, Z. Yu, W. Sun, A. Wang, H. Huang, B. Zou, D. Zhao, X. Li, *Energy Environ. Sci.* **2023**, *16*, 2080.

[S19] H. Cui, L. Huang, S. Zhou, C. Wang, X. Hu, H. Guan, S. Wang, W. Shao, D. Pu, K. Dong, J. Zhou, P. Jia, W. Wang, C. Tao, W. Ke, G. Fang, *Energy Environ. Sci.* **2023**, *16*, 5992.

[S20] H. Liu, J. Dong, P. Wang, B. Shi, Y. Zhao, X. Zhang, *Adv. Funct. Mater.* **2023**, *33*, 2303673.

[S21] Y. An, N. Zhang, Z. Zeng, Y. Cai, W. Jiang, F. Qi, L. Ke, F. R. Lin, S.-W. Tsang, T. Shi, A. K. Jen, H.-L. Yip, *Adv. Mater.* **2024**, *36*, 2306568.

[S22] G. Su, R. Yu, Y. Dong, Z. He, Y. Zhang, R. Wang, Q. Dang, S. Sha, Q. Lv, Z. Xu, Z. Liu, M. Li, Z. Tan, *Adv. Energy Mater.* **2024**, *14*, 2303344.

[S23] H. Guan, W. Zhang, J. Liang, C. Wang, X. Hu, D. Pu, L. Huang, Y. Ge, H. Cui, Y. Zou, G. Fang, W. Ke, *Adv. Funct. Mater.* **2023**, *33*, 2300860.

[S24] R. Wang, J. Zhu, J. You, H. Huang, Y. Yang, R. Chen, J. Wang, Y. Xu, Z. Gao, J. Chen, B. Xu, B. Wang, C. Chen, D. Zhao, W.-H. Zhang, *Energy Environ. Sci.* **2024**, *17*, 2662.

[S25] X. Shen, B. M. Gallant, P. Holzhey, J. A. Smith, K. A. Elmestekawy, Z. Yuan, P. V. G. M. Rathnayake, S. Bernardi, A. Dasgupta, E. Kasparavicius, T. Malinauskas, P. Caprioglio, O. Shargaieva, Y.-H. Lin, M. M. McCarthy, E. Unger, V. Getautis, A. Widmer-Cooper, L. M. Herz, H. J. Snaith, *Adv. Mater.* **2023**, *35*, 2211742.

[S26] Q. Ye, F. Ma, Y. Zhao, S. Yu, Z. Chu, P. Gao, X. Zhang, J. You, Small **2020**, *16*, 2005246.

[S27] R. He, Z. Yi, Y. Luo, J. Luo, Q. Wei, H. Lai, H. Huang, B. Zou, G. Cui, W. Wang, C. Xiao, S. Ren, C. Chen, C. Wang, G. Xing, F. Fu, D. Zhao, *Adv. Sci.* **2022**, *9*, 2203210.

[S28] P. Jia, G. Chen, G. Li, J. Liang, H. Guan, C. Wang, D. Pu, Y. Ge, X. Hu, H. Cui, S. Du, C. Liang, J. Liao, G. Xing, W. Ke, G. Fang, *Adv. Mater.* **2024**, 2400105.

[S29] S. Wang, P. Wang, B. Shi, C. Sun, H. Sun, S. Qi, Q. Huang, S. Xu, Y. Zhao, X. Zhang, *Adv. Mater.* **2023**, *35*, 2300581.

[S30] Y. Zheng, X. Wu, J. Liang, Z. Zhang, J. Jiang, J. Wang, Y. Huang, C. Tian, L. Wang, Z. Chen, C.-C. Chen, *Adv. Funct. Mater.* **2022**, *32*, 2200431.
